# Comparison of In Vitro Antimelanoma and Antimicrobial Activity of 2,3-Indolo-betulinic Acid and Its Glycine Conjugates

**DOI:** 10.3390/plants12061253

**Published:** 2023-03-09

**Authors:** Adelina Lombrea, Alexandra-Denisa Semenescu, Ioana Zinuca Magyari-Pavel, Māris Turks, Jevgeņija Lugiņina, Uldis Peipiņš, Delia Muntean, Cristina Adriana Dehelean, Stefania Dinu, Corina Danciu

**Affiliations:** 1 Department of Pharmacognosy, “Victor Babes” University of Medicine and Pharmacy Timisoara, Eftimie Murgu Square, No. 2, 300041 Timisoara, Romania; adelina.lombrea@umft.ro (A.L.); corina.danciu@umft.ro (C.D.); 2 Research Center for Pharmaco-Toxicological Evaluation, “Victor Babes” University of Medicine and Pharmacy Timisoara, Eftimie Murgu Square, No. 2, 300041 Timisoara, Romania; alexandra.scurtu@umft.ro (A.-D.S.); muntean.delia@umft.ro (D.M.); cadehelean@umft.ro (C.A.D.); 3 Department of Toxicology, “Victor Babes” University of Medicine and Pharmacy Timisoara, Eftimie Murgu Square, No. 2, 300041 Timisoara, Romania; 4 Institute of Technology of Organic Chemistry, Faculty of Materials Science and Applied Chemistry, Riga Technical University, P. Valdena Str. 3, LV-1048 Riga, Latvia; maris.turks@rtu.lv (M.T.); jevgenija.luginina@rtu.lv (J.L.); sales@nstchemicals.com (U.P.); 5 Nature Science Technologies Ltd., Rupnicu Str. 4, LV-2114 Olaine, Latvia; 6 Department of Microbiology, “Victor Babes” University of Medicine and Pharmacy Timisoara, Eftimie Murgu Square, No. 2, 300041 Timisoara, Romania; 7 Department of Pedodontics, Faculty of Dental Medicine, “Victor Babes” University of Medicine and Pharmacy Timisoara, 9 No., Revolutiei Bv., 300041 Timisoara, Romania; dinu.stefania@umft.ro; 8 Pediatric Dentistry Research Center, Faculty of Dental Medicine, “Victor Babes” University of Medicine and Pharmacy Timisoara, 9 No., Revolutiei Bv., 300041 Timisoara, Romania

**Keywords:** 2,3-indolo-betulinic acid, glycine conjugates, melanoma, antibacterial

## Abstract

Malignant melanoma is one of the most pressing problems in the developing world. New therapeutic agents that might be effective in treating malignancies that have developed resistance to conventional medications are urgently required. Semisynthesis is an essential method for improving the biological activity and the therapeutic efficacy of natural product precursors. Semisynthetic derivatives of natural compounds are valuable sources of new drug candidates with a variety of pharmacological actions, including anticancer ones. Two novel semisynthetic derivatives of betulinic acid—N-(2,3-indolo-betulinoyl)diglycylglycine (BA1) and N-(2,3-indolo-betulinoyl)glycylglycine (BA2)—were designed and their antiproliferative, cytotoxic, and anti-migratory activity against A375 human melanoma cells was determined in comparison with known N-(2,3-indolo-betulinoyl)glycine (BA3), 2,3-indolo-betulinic acid (BA4) and naturally occurring betulinic acid (BI). A dose-dependent antiproliferative effect with IC_50_ values that ranged from 5.7 to 19.6 µM was observed in the series of all five compounds including betulinic acid. The novel compounds BA1 (IC_50_ = 5.7 µM) and BA2 (IC_50_ = 10.0 µM) were three times and two times more active than the parent cyclic structure B4 and natural BI. Additionally, compounds BA2, BA3, and BA4 possess antibacterial activity against *Streptococcus pyogenes* ATCC 19615 and *Staphylococcus aureus* ATCC 25923 with MIC values in the range of 13–16 µg/mL and 26–32 µg/mL, respectively. On the other hand, antifungal activity toward *Candida albicans* ATCC 10231 and *Candida parapsilosis* ATCC 22019 was found for compound BA3 with MIC 29 µg/mL. This is the first report of antibacterial and antifungal activity of 2,3-indolo-betulinic acid derivatives and also the first extended report on their anti-melanoma activity, which among others includes data on anti-migratory activity and shows the significance of amino acid side chain on the observed activity. The obtained data justify further research on the anti-melanoma and antimicrobial activity of 2,3-indolo-betulinic acid derivatives.

## 1. Introduction

According to the current estimates, the plant kingdom has about 250,000 species, of which only about 10% have been systematically researched for their phytochemical constituents and therapeutic potential [1]. These plant species have been assigned with a large number of phytoconstituents endowed with a plethora of pharmacological properties, including anti-diabetic, antimicrobial, hepatoprotective, anti-inflammatory, antimalarial, anti-ageing, immunomodulator, antioxidant, antihypertension, and anticancer activities, etc., [2,3,4]. In this line, natural product research and development may play a critical role in the emergence of novel drug candidates. Modern drug development approaches have abandoned the use of entire plant extracts in place of individual compound-based therapy [5]. Pentacyclic triterpenoids (PTs) are naturally occurring in the fruit peel, leaves, and bark of plants (e.g., the peel of apples, leaves of eucalyptus, and birch bark) [6]. Triterpenoids are modified triterpenes that may be produced in plants by cyclizing a squalene intermediate containing six isoprene units [7]. From a biological perspective, the majority of notable triterpenoid compounds are triterpenoids derived from ursane (ursolic acid, uvaol, and α-amyrin), oleanane (oleanolic acid, maslinic acid, erythrodiol, and β-amyrin), and lupane (lupeol, betulin, betulinic acid, betulonic acid) skeleton [6]. PTs have been assigned with a diverse spectrum of bioactivities, including, antiangiogenic, anti-inflammatory, antiviral, antioxidant, anticancer, antidiabetic, hypolipidemic, antibacterial, hepatoprotective, and cardioprotective ones [8,9,10,11,12,13,14]. Compelling research studies have addressed the cytotoxicity of PTs on a variety of cancer cells, demonstrating their non-toxic effects on normal cells [15,16]. For instance, these plants’ secondary metabolites and their derivatives have been shown to be responsible for triggering cell apoptosis via the intrinsic pathway by altering the permeability of the mitochondrial membrane and increasing the release of cytochrome c [17,18,19]. It has been found that betulinic acid hinders proliferation in a variety of cell lines, including human gastric cancer cell lines (MGC-803, SGC-7901), human prostate cancer cell lines (PC3, LNCaP), lung cancer cell line (A549), human breast cancer cell line (MCF-7), human liver cancer cell line (HepG-2), human ovarian cancer cell line (SK-OV-3), human melanoma cancer cell lines (A375, SK-MEL-2), and others [17,18,19,20,21].

The triterpenic scaffold yields poor water solubility, increased lipophilicity and suboptimal bioavailability, hence limiting its therapeutic use [13]. To address this hurdle, the synthesis of semisynthetic derivatives obtained from PTs has become a key method for improving pharmacological activities such as targeted selectivity or enhanced bioavailability [22]. For example, oleanolic acid served as the backbone for chemical modifications to produce more bioactive derivatives, such as 2-cyano-3,12-dioxoleana-1,9-dien-28-oic acid (CDDO) and its equivalent methyl (CDDO-Me) and imidazole (CDDO-Im) esters [23,24]. According to in vitro and in vivo investigations, CDDO, CDDO-Me, and CDDO-Im have the potential to be effective chemotherapeutics by inhibiting proliferation, angiogenesis, and triggering apoptosis in pancreatic and colon cancer cells. The first phase I clinical study of CDDO-Me was undertaken in advanced solid tumor and lymphoma patients to assess the dose-limiting toxicity (DLT) and maximum dose (MTD). CDDO-Me has shown a potential anticancer effect, and it has additionally been observed that it improves kidney function in individuals with type 2 diabetes and persistent kidney disease. After phase I, individuals with moderate to severe CKD and type 2 diabetes have participated in a phase II study employing CDDDO-Me. Improvements in renal function have been recorded [24,25,26,27]. Growing research is shedding light on preclinical studies performed on betulonic acid and its chemical derivatives [28]. Betulonic acid is an outstanding pentacyclic triterpenoid belonging to the lupane class which can be extracted from the Chinese sweet gum (*Liquidambar formosana* Hance) fruit [29], blackthorn (*Acacia mellifera* L.) bark [30], aerial roots of Chinese banyan (*Ficus microcarpa* L. f.) [31], etc. Additionally, it may be yielded through selective oxidation of betulin [32]. The available scientific literature reveals that betulonic acid and its derivatives exhibit antiviral [33,34], antimicrobial [35,36], anti-human cytomegalovirus [37], anti-inflammatory [38,39], antioxidant [40], hepatoprotective [39,41], immunostimulant [42], and anticancer properties [43,44]. Betulonic acid and its chemical derivatives can be put forth to therapeutical applications in the oncologic field since it is effective in suppressing the proliferation of a variety of tumor cell lines, including A431 (skin carcinoma), A2780 (ovarian carcinoma), MCF-7 (breast adenocarcinoma), A375 (malignant melanoma), PC3 (prostate cancer), MGC-803 (gastric cancer), Bcap-37 (breast adenocarcinoma) [45,46]. Recent studies have shown that among these chemical derivatives, those with heterocyclic scaffolds containing nitrogen plays a significant role. The literature describes that heterocyclic compounds containing nitrogen are often more stable and have a higher bioavailability. Additionally, heterocyclic derivatives exhibit strong absorption bands under UV light irradiation, which facilitates their identification in a variety of in vivo assays and subsequent clinical trials [47,48]. Among the semi-synthetic compounds containing a fused heterocylce in the triterpenoid structure 2,3-indolo-betulinic acid and its derivatives stand out with enhanced antiproliferative activity and selectivity against the malignant cell lines [49,50].

On a worldwide scale, cancer has been identified as a primary cause of mortality, and a 70% incidence increase is estimated in the next two decades [51]. While recent breakthroughs in precision medicine using targeted therapies are encouraging, the histological and molecular heterogeneity of cancer cells, as well as the enormous mutational loads, which contribute to resistance and treatment failure, continue to represent significant obstacles. [52]. In the current scenario, an extensive number of ongoing preclinical studies have reported valuable insights on the anticancer activity of plants’ secondary metabolites, especially, pentacyclic triterpenes [8,28,53,54,55,56]. Among the highly aggressive forms, malignant melanoma can be mentioned. This form of cancer takes over 20,000 lives each year in Europe, due to its growing prevalence, increased mortality. Melanocytes originate from the neural crest and hence express a large number of signaling molecules and factors that facilitate migration and metastasis [57]. Melanoma is not limited to the skin; it may also occur in the ocular, mucosal, or leptomeningeal cavities. It is caused by a combination of environmental (particularly UVB radiation) and genetic susceptibility, melanocytic and abnormal nevi, personal history, and occupational risk factors [58,59,60]. Until now, therapeutic options for melanoma have included surgery, immunotherapy, targeted therapy, chemotherapy, and radiation therapy [61]. While surgery may be used to treat early stage melanoma, surgery alone is not adequate to treat metastatic melanoma [62]. Given the high impact of this malignancy, phytocompounds are undergoing extensive research as possibly more effective and safer targeted therapy for melanoma [63].

Due to the alarming growth of antimicrobial resistance as well as the difficulty in developing novel antimicrobial medications, alternative techniques to manage bacterial infections are urgently required, and natural and semisynthetic pentacyclic triterpenoids provide a broad range of uses in this field [64,65]. Recent research has shown that a series of 34 novel betulinic, ursolic, and oleanolic acid derivatives bearing amino and guanidinium groups on the C-3 and C-28 side chains inhibit the growth of methicillin-resistant *Staphylococcus aureus* (MRSA) [minimum inhibitory concentrations (MIC) ≤ 0.25 µg/mL] which is superior to that of vancomycin (MIC = 1 µg/mL) [66]. With MIC values of 25 and 50 µM, oleanonic acid amide with diethylenetriamine has been proven to be effective against *Escherichia coli* and *Pseudomonas aeruginosa* [67].

To the best of our knowledge, the anti-melanoma activity of 2,3-indolo-betulinic acid has been studied only very briefly and several important melanoma cell lines such as A375 have been omitted from that research [49]. On the other hand, the antimicrobial activity of 2,3-indolo-betulinic acid and its derivatives has not been reported at all. 

The primary aim of this study is to assess the impact of the size of the glycine side chain on the antiproliferative, cytotoxic, and anti-migratory activities against A375 human melanoma cell line of preexisting 2,3-indolo-betulinic acid (BA4) and N-(2,3-indolo-betulinoyl)glycine (BA3) versus newly designed N-(2,3-indolo-betulinoyl)glycylglycine (BA2) and N-(2,3-indolo-betulinoyl)diglycylglycine (BA1) possessing two and three glycine moieties, respectively. As a reference benchmark, naturally occurring betulinic acid was employed. Furthermore, the second objective of the study is to determine the antimicrobial activity of the title compounds. From the structure–activity relationship point of view, our findings clearly show the enhanced anti-melanoma activity of newly designed 2,3-indolo-betulinic acid conjugate containing diglycylglycine fragment. Regarding antimicrobial effects, the insertion of glycine moieties has shown significant promise.

## 2. Results

### 2.1. Chemical Synthesis of the Compounds

**Figure 1:**
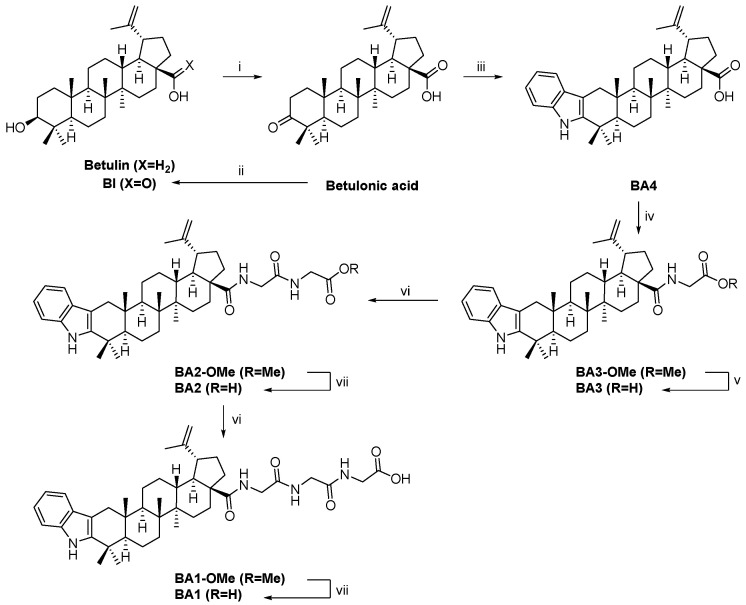
Synthetic route for the preparation of triterpenoid derivatives. Reagents and conditions: (i): Khlebnicova 2017; (ii): Levdanskii 2015; (iii): Khusnutdinova 2019; (iv): Kumar 2008; (v): Mukherjee 2008; (vi): acid, EDC·HCl, HOBt, DIPEA, HCl·H-Gly-OMe, DCM, RT, 24 h; (vii): ester, NaOH, H_2_O/MeOH, 45 °C, 2 h.

The reaction conditions and synthesis methodologies utilized to generate betulinic acid (BI), betulinic acid—N-(2,3-indolo-betulinoyl)diglycylglycine (BA1), N-(2,3-indolo-betulinoyl)glycylglycine (BA2), N-(2,3-Indolo-betulinoyl)glycine (BA3), and 2,3-Indolo-betulinic acid (BA4) are displayed in Figure 1. Good yields were obtained for the previously documented compounds BA3 and BA4 (>70%), as well as for the newly synthesized derivatives BA1 and BA2 (>90%). Using ^1^H and ^13^C NMR spectroscopy, the structures of all produced compounds were confirmed. The NMR spectra of betulonic acid, BI, BA3, and BA4 matched those previously described in the literature [50,68,69,70,71]. All NMR spectral data are provided in the Supplementary Figures S1–S12 attached to the current publication.

### 2.2. Cell Viability Assay

**Figure 2:**
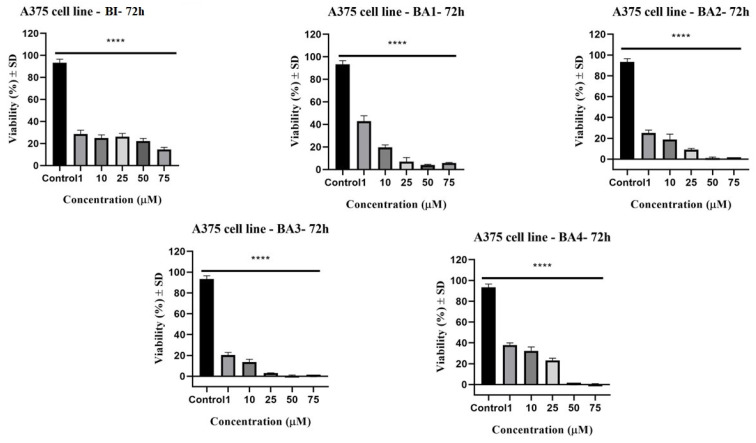
Viability of A375 human melanoma cells following 72 h stimulation with BI, BA1, BA2, BA3, and BA4 at concentrations of 1, 10, 25, 50, and 75 µM. The study results are reported as a percentage (%) of cell viability in comparison to the control cells. One-way ANOVA followed by Dunnett’s multiple comparison post-test was used to compare the groups. A *p*-value ≤ 0.05 was regarded as statistically significant (**** *p* ≤ 0.0001 vs. control).

Treatment of A375 melanoma cells with BI, BA1, BA2, BA3, and BA4 in dimethyl sulfoxide (DMSO) solution (1, 10, 25, 50, and 75 µM) has validated our hypothesis; hence, 2,3-indolo-betulinic acid derivatives seem to be prospective anticancer agents. As captured in Figure 2, betulinic acid and 2,3-indolo-betulinic acid derivatives displayed antiproliferative activity in a dose-dependent fashion. Each of the screened compounds exhibited a statistically significant decrease in cell viability. Following 72 h incubation, the IC50 values for these chemicals ranged from 5.7 to 19.2 µM (Table 1). Additionally, among the synthetic 2,3-indolo-betulinic acid derivatives, compound BA1 was the most potent one. This derivative was about three-fold more active than betulinic acid (IC_50_ = 5.7 µM vs. IC_50_ = 19.2 µM).

### 2.3. Evaluation of the Cytotoxic Potential by Lactate Dehydrogenase (LDH) Assay

**Figure 3:**
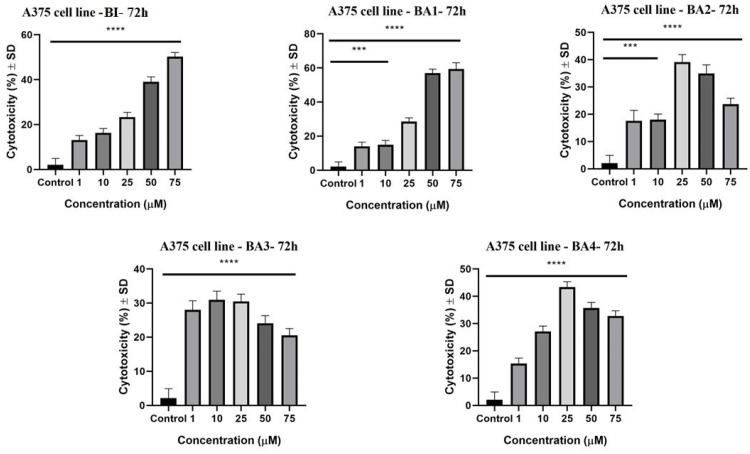
The cytotoxic activity of BI, BA1, BA2, BA3, and BA4 at concentrations of 1, 10, 25, 50, and 75 µM on A375 cells after 72 h of incubation. The data are presented as a cytotoxicity percentage (%) ± SD compared to control cells. A one-way ANOVA test and Dunnett’s multiple comparison post-test were used to compare the groups (*** *p* ≤ 0.001; **** *p* ≤ 0.0001 vs. control).

The LDH assay was employed to evaluate the cytotoxicity of betulinic acid and 2,3-indolo-betulinic acid derivatives on human melanoma cells (A375). The LDH test measures the amount of lactate dehydrogenase released into the medium, which demonstrates cell death and membrane disruption [72]. Following 72 h of stimulation, the cytotoxicity evaluation revealed that betulinic acid along with the four 2,3-indolo-betulinic acid derivatives have promoted lactate dehydrogenase release in a concentration-dependent fashion (Figure 3). Among the derivatives, BA1 at the maximal concentration of 75 µM, exhibited the most substantial LDH release (59.3% ± 2.3) compared with betulinic acid (50.2% ± 1.8). Additionally, at the same dose, the LDH release of cells treated with BA1 and the low cell viability rate were directly correlated. In addition, among the glycine conjugates, the one with a diglycylglycine side chain displayed the highest cytotoxic effect.

### 2.4. Anti-Migratory Activity Evaluation Using the Scratch Assay Method

**Figure 4:**
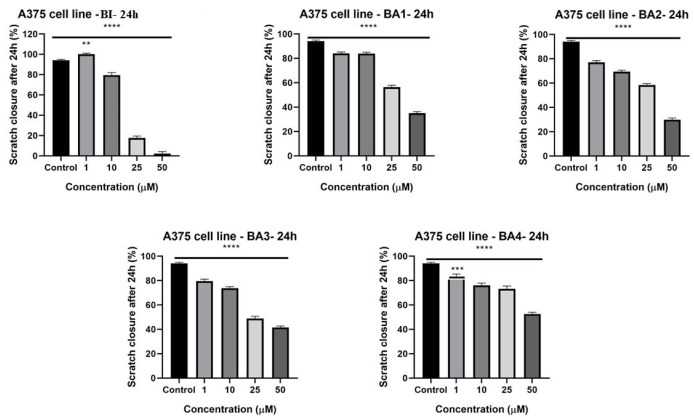
Images depicting the migratory potential of A375 cells after treatment with BI, BA1, BA2, BA3, and BA4 for 24 h. The bar graphs show the percentage of wound closure relative to the baseline surface after 24 h. The results are shown as the mean values ± SD of three separate trials carried out in triplicate. Applying the one-way ANOVA analysis and then Dunnett’s multiple comparisons post-test (** *p* ≤ 0.01; *** *p* ≤ 0.001; **** *p* ≤ 0.0001 vs. control) were used to determine the significant differences between the control and treated groups.

Since malignant melanoma cells exhibit significant rates of metastasis, a wound-healing assay has been used to examine the possible anti-migratory properties of betulinic acid and 2,3-indolo-betulinic acid derivatives. According to Figure 4, all of the evaluated compounds reduced the ability of A375 cells to migrate in a dose-dependent manner in comparison to the control cells (*p* ≤ 0.0001). At higher concentrations (25 µM and 50 µM), tested compounds have significantly hampered the cells’ migration (73.2%−2.1%). Treatment of cells with 25 µM BI significantly inhibited cell migration, resulting in scratch closure rates of 17.5%. BA1 and BA2 (50 µM), the two novel derivatives, have also demonstrated potent anti-migratory effects with wound healing rates of 35% and 30%, respectively. BA4 was associated to the weakest increase in cell migration, which was closely tied with its poor cytotoxic profile. Additionally, it can be seen from the images from Supplementary Figures S13–S15, that following 24 h of stimulation with the tested compounds, at larger concentrations, the cells displayed apoptotic features by altering their form and morphology, accompanied by cell disintegration (see Supplementary Figures S13–S15). These details indicate that the 2,3-indolo-betulinic acid and its glycine conjugates had a cytotoxic influence on the human melanoma cancer cells.

### 2.5. Antimicrobial Activity Assays

The in vitro antimicrobial activity of betulinic acid and 2,3-indolo-betulinic acid and its derivatives was determined on Gram-positive and Gram-negative bacterial strains, as well as *Candida* spp. The findings of the disk diffusion method (represented as the diameter of the inhibition zone) and the dilution method [reported as the minimum inhibitory concentration (MIC) and minimum bactericidal concentration (MBC) or minimum fungal concentration (MFC)] are summarized in Table 2. The MIC value was established as the lowest concentration at which no visible growth occurred, whereas MBC was regarded as the lowest dose of the tested derivatives that decreases the amount of surviving bacteria by at least three logs.

Three out of four derivatives (BA2, BA3, BA4) effectively inhibited the growth of Gram-positive bacteria (*Streptococcus pyogenes* ATCC 19615 and *Staphylococcus aureus* ATCC 25923), with MIC in the range of 13–33 µg/mL, whereas betulinic acid was inactive against all microbial strains. Noteworthy, the 2,3-indolo-betulinic acid BA4 displayed the best antibacterial activities against *Streptococcus pyogenes* (MIC = 13 µg/mL), followed by *Staphylococcus aureus* (MIC = 26 µg/mL). On the other hand, the tested compounds did not have the same effects on Gram-negative *Pseudomonas aeruginosa* ATCC 27853 and *Escherichia coli* ATCC 25922. One explanation is that the outer membrane, which is typical of Gram-negative bacteria, may function as a targeted permeability barrier that prevents these chemicals from exerting injuries. In terms of antifungal action, *Candida albicans* ATCC 10231, *Candida parapsilosis* ATCC 22019 were highly sensitive to 2,3-indolo-betulinic acid—monoglycine conjugate BA3. Similar inhibition diameters were observed for the aforementioned compound and fluconazole (16 mm).

## 3. Discussion

The semisynthetic modifications of pentacyclic triterpenes provide a broad variety of functionalities, from increasing potency and/or selectivity to optimizing pharmacokinetic parameters [73]. One of the several nitrogen heterocyclic compounds present in nature, the indole ring is often present in plant hormones such as tryptophan and auxins, serotonin or 5-hydroxytryptamine, and melatonin [74]. The formation of fused cycles between indole and PTs is an efficient method for identifying and developing new anticancer agents. A single molecule comprising one or more pharmacophores with unique molecular pathways may improve the desirable features of the combined components and prevent drug resistance [75].

This is the first study to demonstrate the dependence of antiproliferative, cytotoxic, anti-migratory, and antimicrobial properties of 2,3-indolo-betulinic acid derivatives on the number of glycine fragments attached to the triterpenoid core. Our findings demonstrate a dose-dependent reduction in the viability of A375 melanoma cells after 72 h post-incubation with tested compounds (Figure 2). When compared to betulinic acid, three of the four 2,3-indolo-betulinic acid derivatives showed higher antiproliferative activity against melanoma cells, with IC_50_ values for BA1, BA3, and BA2 being 5.7, 10.0, 13.7 vs. 19.2 µM, respectively. The most promising derivative was *N*-(2,3-indolo-betulinoyl)diglycylglycine BA1 as reflected by the obtained IC_50_ value of 5.7 µM. At maximum concentration (75 µM), BA1 inhibits cell viability to 5.8%. Noteworthy, at a lower concentration such as 10 µM, the tested derivatives have significantly decreased the percentage of viable cancer cells: for BA1, it was 19.6%, for BA2 it was 18.7%, for BA3 it was 13.6%, and for BA4 it was 32.3%. Conversely, at the same dosage of 10 µM, betulinic acid reduced the cell viability only by 25.04%. As captured in Figure 2, at higher concentrations of 50 and 75 µM BA2, BA3, and BA4 have displayed cell viability in the range of −0.4% and 5.8%. We hypothesized that a variety of parameters, such as metabolic and energy disturbances, modifications in the performance of oxidoreductases, endo-/exocytosis, and intracellular transport, might have a major impact on MTT reduction. Adaptive metabolic and mitochondrial reprogramming may contribute to the underestimation of cell viability by the MTT analysis in cells stimulated with tested compounds [76]. Regarding the antiproliferative profile of betulinic acid against the A375 cell line, our results are comparable to those of other researchers. For instance, in the sulforhodamine B-assay performed by Liebscher et al., on A375 cells, following 96 h of incubation, betulinic acid displayed an IC_50_ value of 13.3 µM [77]. Wróblewska-Łuczka et al. revealed in a study published this year that betulinic acid has an antiproliferative effect on A375 melanoma cells after 72 h of incubation (IC_50_ = 15.94 µM) [78]. Khusnutdinova et al. examined the potential cytotoxic effect of 2,3-indolo-betulinic acid (100 µM) against a panel of melanoma cell lines (LOX IMVI, MALME-3M, M14, MDA-MB-435, SK-MEL-2, SK-MEL-28, SK-MEL-5, UACC-257, and UACC-62). LOX IMVI melanoma cells were the most responsive to the tested derivative after 48 h of stimulation, with a viability percentage of 44%, followed by SK-MEL-2 cells, with a viability percentage of 57% [49]. Conversely, in our investigation, at 72 h after stimulation with 2,3-indolo-betulinic acid (75µM), the cellular viability of A375 melanoma cells dropped to 0.17%. Jeong et al. synthesized C-28 amino acid conjugates, which elevated the selective toxicity against human melanoma (MEL-2) as well as the aqueous solubility of betulinic acid. Converting methyl ester of glycine conjugates to the equivalent free acid conjugates enhanced their cytotoxicity toward MEL-2 (IC_50_ = from 10.2 to 4.2 µg/mL). The authors concluded that the free acid of glycine displayed excellent water solubility while retraining the specific cytotoxicity of the original molecule, betulinic acid [79]. Kumar et al. have synthesized 2,3-annealed indole derivatives of betulinic and betulonic acid by Fisher reaction and conducted an extensive SARS experiment. Approximately 30 indole derivatives were examined on a panel of eight cancer cell lines, excluding melanoma. According to the results, the insertion of an indol heterocycle to a terpene increases its cytotoxicity when both the N-1 and C-28 sites remain unoccupied. However, the most active derivative, which was conjugated with glycine via C-28, constituted an exception to this hypothesis (IC_50_ values between 0.67 and 3.53 µg/mL for MIAPaCa, PA-1, and A549 cancer cell lines) [50]. The migratory potential of cancer cells plays a significant role in cancerogenesis and dissemination, and identifying drugs that may inhibit this effect is a key process in revealing novel pathways involved in the progression of cancer [80]. The influence of BI, 2,3-indolo-betulinic acid, and its derivatives on cellular proliferation and migration was assessed using scratch assay, a method similar to wound healing. The A375 control cells exhibited migratory propensity by filling the wound area after 24 h, whereas cells treated with examined substances exhibited a suppression of the cell process of migration. As seen in Figure 4, overall, the newly synthesized compound BA3, has significantly impeded the migratory capacity of human melanoma cells. Furthermore, the cells in the control group had a spindle form with a distinct outline. In the treated groups, the number of A375 cells considerably dropped, especially in the group treated with a high concentration of the tested compounds, and the cells developed a round shape, which is characteristic of apoptotic cells. The group of Coricovac et al., have also published same results for BI. Starting at 25 µM, BI generated substantial alterations in the morphology of A375 melanoma cells, including round shape and very poor adhesion to the plate [81].

In another study, Yang et al. thoroughly investigated the cytotoxic potential of 25 betulonic acid derivatives against malignant melanoma A375 [46]. According to the MTT assays, the addition of amino groups at the C-28 position has resulted in a strong anticancer activity that is two to eight times more effective than betulonic acid with IC_50_ values between 6.8 and 17 µM. While substituted alkyl carboxylic acids displayed low anticancer activity (IC_50_ values > 20 µM), substituted benzoic acids exhibited moderate to high cytotoxic activity (IC_50_ values from 4.5 µM to 9.3 µM). The efficacy was diminished when the 28-COOH was solely replaced with a dibromoalkane (IC_50_ values beyond 20 µM) [46]. According to extensive preclinical research in the area of oncology, the placement of piperazine fragments into the C2, C3, and C28 sites of triterpenoids, resulted in a significant degree of inhibitory effects toward several human tumor cell lines [82,83,84,85]. Furthermore, piperazine moieties are advantageous for enhancing the pharmacokinetic parameters, acid-base balance factors, and lipid–water partition coefficient of medicines [86]. Giniyatullina et al. characterized the interaction of betulonic acid with aldehydes at C2 to be encouraging since it produced derivatives containing 4-pyridinoylidene and furfurylidene fragments with strong anticancer activity against NCI-60 cell line panel. Screening of the cytotoxicity has been carried out at a concentration of 10 µM, employing the Sulforhodamine B assay. The findings revealed the prospective therapeutic efficacy of 2-(furfurylidene)-betulonic acid, as shown by 56 occurrences of cancer cell lethality ranging from −98.18 % to 31.8 %. The cytotoxicity of 2-(furfurylidene)-betulonic acid against nine melanoma cell lines (LOX IMVI, MALME-3M, M14, MDA-MB-435, SK-MEL-2, SK-MEL-28, SK-MEL-5, UACC-257, and UACC-62) was remarkable, with the highest antiproliferative activity observed in UACC-62 (−97.61%), followed by MALME-3M (−82.26%), and SK-MEL-2 (−75.96%). In contrast, the 2-(pyridinoylidene)-betulonic acid has elicited the strongest antiproliferative effect on LOX IMVI (−9.49%), followed by SK-MEL-5 (89.4% inhibitory rate) and MDA-MB-435 (80.19% inhibitory rate) [87]. 

The LDH assessment provides the evaluation of cell damage resulting from the release of the enzyme lactate dehydrogenase by lysed cells following membrane rupture. In our work, there was a clear association between the cytotoxicity of the investigated compounds and the antiproliferative assay (Figure 3). For BA2, BA3, and BA4 compounds, the maximal cytotoxic effect against A375 cells was achieved at 25 µM concentration (BA2: 39.13% ± 2.7; BA3: 30.4% ± 2.1; BA4: 43.36% ±1.9), whereas at larger concentrations (75 µM), the cytotoxic rate slightly decreased (BA2: 23.69% ± 2.2; BA3: 20.54% ± 2.0; BA4 32.8% ± 1.8). This tendency was previously observed in A375 human melanoma cell line investigations with substances that might trigger cell cycle arrest [88,89]. Given the fact that cells no longer display proliferative characteristics, the quantity of LDH that may be produced from cells will be fairly low, hence diminishing the cytotoxic impact of the sample [72,89]. Concerning the cytotoxicity of betulinic acid, our findings of the LDH assay correspond to those reported by Wróblewska-uczka et al. After 72 h of incubation with 40 µM betulinic acid, the LDH release rate of human melanoma cells A375 was around 45% [78]. 

In natural products, pentacyclic triterpenoids are plentiful and function as crucial substrates for the synthesis of numerous major bioactive components. By linking a triterpenoid scaffold with amines, amino acids, and polyamines, a multitude of anticancer, antibacterial, and antiviral molecules were established [67]. The correlations between the chemical structure and antibacterial activity of triterpenes have been documented in the current literature. Furthermore, it has been noted that the chemical structure of the substituents has an impact on bactericidal activity [90]. In this study, three of four 2,3-indolo-betulinic acid derivatives displayed comparable antibacterial activity against *Streptococcus pyogenes* and *Staphylococcus aureus*, with inhibition zone diameters between 16 and 17 mm, whereas Gram-negative bacteria were resistant (Table 2). These results are consistent with those reported in the literature. The antibacterial activity of the studied derivatives may be attributed to the fact that conjugating a water-soluble amino acid such as glycine may enhance their water solubility, hence improving their biological activity [79]. Gram-negative bacteria have been shown to be more antibiotic-resistant compared to Gram-positive bacteria owing to their external lipidic membrane, which inhibits the passage of chemicals to the periplasm [91]. On the other hand, betulinic acid was ineffective against *Streptococcus pyogenes, Staphylococcus aureus, Escherichia coli Pseudomonas aeruginosa, Candida parapsilosis, and Candida albicans.* On this topic, the literature provides contradictory evidence. For instance, according to some publications, betulinic acid is inactive against *Staphylococcus aureus*, *Bacillus subtilis*, *Escherichia coli, Enterococcus faecalis, Pseudomonas aeruginosa,* and *Candida albicans* (minimum inhibitory concentration (MIC) values above 256 mg/L), validating our conclusions [92,93]. This inactivity of betulinic acid is due to its limited solubility in water and, to a limited extent, in typical organic solvents [94,95,96]. Other investigations, conversely, have indicated that betulinic acid, has poor activity (MIC ≥ 128 mg/mL) against *Bacillus cereus*, *Klebsiella pneumoniae Enterococcus fecalis*, *Escherichia coli*, *Listeria monocytogenes*, *Pseudomonas aeruginosa*, *Salmonella enterica*, and *Staphylococcus aureus* [91,97].

It is well-documented that the incorporation of nitrogen-containing substituents into a triterpenoid skeleton contributed to the emergence of a variety of anticancer, antibacterial, and antiviral drugs. In this regard, Kazakova et al. have newly synthesized betulonic acid diethylenetriamine conjugate which has been evaluated for antimicrobial activity. At a concentration of 32 µg/mL, the derivative was partially active against *Staphylococcus aureus* ATCC 43300 with an inhibition value of 72% and the fungal strain *Cryptococcus neoformans* ATCC 208821 with a 63% inhibition rate. The derivative was ineffective when tested at the same concentration against *Escherichia coli* ATCC 25922, *Klebsiella pneumonia* ATCC 700603, *Acinetobacter baumannii* ATCC 27853, *Pseudomonas aeruginosa* strain 19606, and *Candida albicans* ATCC 90028 [98]. In recent years, drug development based on piperazine has received substantial interest. According to studies, the insertion of a piperazine moiety may yield significant enhancements in the bioactivity of drugs [85]. The antibacterial potential of introducing a piperazine moiety into triterpene acid compounds was reported by Kazakova et al. N-methylpiperazinyl amide of betulonic acid has elicited the highest inhibitory activity against *Staphylococcus aureus*. At a dose of 80 mg/mL, the derivative prevented the development of 50% of *Staphylococcus aureus* colonies and reduced the quantity of germs to 1 × 10^3^ CFU/mL, whereas at a concentration of 90 mg/mL, it displayed bacteriostatic activity, and at a concentration of 100 mg/mL, it exhibited bactericidal effect [35].

## 4. Materials and Methods

### 4.1. Chemistry

#### 4.1.1. Instruments

Prior to use, the solvents for the reactions were dried using conventional drying agents and subsequently distilled. All bought (Fluka, Aldrich, St. Louis, MO, USA) chemicals were used as supplied. All reactions were monitored using thin-layer chromatography (TLC) on E. Merck Kieselgel 60 F254 and a UV light for visualization. Silica gel was employed for column chromatography (60 Å, 40–63 μm, ROCC, Sart Eustache, Belgium). Optical rotations were measured at 25 °C on a *Anton Paar MCP 500* polarimeter (1 dm cell) using a sodium lamp as the light source (589 nm). Fourier transform infrared (FT-IR spectrometer, Spectrum 100, PerkinElmer, Waltham, MA, USA) spectra were recorded with Universal ATR Sampling Accessory (UATR, PerkinElmer, Waltham, MA, USA). Spectra were obtained at 4 cm^−1^ resolution co-adding ten scans over a range of wavenumbers from 650 cm^−1^ to 4000 cm^−1^. ^1^H and ^13^C NMR spectra were recorded on a Bruker 500 MHz, in CDCl_3_ or [D_6_]DMSO at 25 °C. Chemical shifts (δ) values are reported in ppm. The residual solvent peaks are used as internal reference (CDCl_3_ 7.26 ppm, [D_6_]DMSO 2.50 ppm for ^1^H NMR, CDCl_3_ 77.16 ppm, [D_6_]DMSO 39.52 ppm for ^13^C NMR), s (singlet), d (doublet), t (triplet), q (quartet), m (multiplet); *J* in hertz. High-resolution mass spectra (ESI) were performed on Agilent 1290 Infinity series UPLC connected to Agilent 6230 TOF mass spectrometer (calibration at m/z 121.050873 and m/z 922.009798).

#### 4.1.2. Synthesis

General procedure I for an amino acid ester synthesis: A zero-degree temperature was applied to a solution of acid (1 equiv.), EDC·HCl (1.2 equiv.), and HOBt (1.2 equiv.) in DCM (80 mL). To this solution, DIPEA (4.4 equiv.) was added, and then HCl·H-Gly-OMe (1.2 equiv.). The solution was warmed to room temperature and agitated for twenty-four hours. Following introducing 200 mL of DCM to the reaction, the mixture was transported to the separatory funnel. The organic layer was rinsed with 50% NaHCO_3_ aq. (3 × 50 mL), brine (50 mL), dried on Na_2_SO_4_, before the solvent was evaporated at reduced pressure. Column chromatography was employed to purify the raw material. 

General procedure II of ester hydrolysis: A solution of ester (1 equiv.), NaOH (2 equiv.), water (35 equiv.) in MeOH (100 mL) was heated at 45 °C for 2 h. When TLC showed that the reaction was complete and under low pressure, the solvent was eliminated. Then DCM (200 mL) was added to the reaction and it was transferred to the separation funnel. The organic layer was rinsed with 5% HCl aq. solution (3 × 50 mL) and brine (50 mL), and dried over Na_2_SO_4_; the solvent was then extracted under reduced pressure to produce acid.

Betulonic acid: Betulin (75 g, 169.4 mmol, 1 equiv.) was placed in a 2 L reactor and dissolved in acetone (150 mL) at −12 °C. Freshly prepared Jones reagent (prepared from CrO_3_ (81.3 g, 812.88 mmol, 7.2 equiv.) in H_2_O (110 mL) and H_2_SO_4_ (156.1 g, 1.592 mol, 14.1 equiv.) in H_2_O (280 mL)) was added dropwise from an additional funnel to the reaction solution over 1 h while controlling the temperature at −10 °C. After 1 h, stirring was continued at 0 °C temperature for 16 h, and then iPrOH (62 mL) was slowly added. The reaction mixture was filtered, and the filtrate evaporated under reduced pressure. The solid residue after filtration was rinsed with DCM (3 × 250 mL) and combined with the evaporated filtrate. Then the reaction was placed in a separating funnel, and the separated organic phase was mixed with H_2_O (250 mL) and saline water (4 × 50 mL) and then dried with anhydrous Na_2_SO_4_. Under decreased pressure, the solvent was evaporated to yield 62 g (80%) of betulonic acid. Crude betulonic acid was purified by crystallization of its cyclohexylamine salt, and its ^1^H and ^13^C NMR spectra match those reported in the literature [68,99].

BI: Betulonic acid (20 g, 439.8 mmol, 1 equiv.) was solubilized in 400 mL of MeOH and chilled to 0 °C. NaBH_4_ (4.11 g, 109.9 mmol, 2.5 equiv.) was slowly added, and the reaction was agitated for four hours at room temperature. The liquid was then filtered by 12 g of Al_2_O_3_ and evaporated under decreased pressure. Solid residue was dissolved in MeOH (360 mL), then charcoal (12 g) was added and the mixture was heated under reflux for 1 h. After the filtration (while hot), the solid residue was dried in 70 °C for 12 h. Yield 10.81 g, 54%. White solid powder. ^1^H and ^13^C NMR spectra match those reported in literature [69]. Table 3 contains the trivial name and structural formula of BI.

BA1: According to general method II, compound BA1 was prepared from BA1-OMe (1.25 g, 1.75 mmol, 1 equiv.), NaOH (0.14 g, 3.5 mmol, 2 equiv.), water (1.1 mL, 61.4 mmol, 35 equiv.). Yield 1.12 g, 92%. Yellow solid powder. [α]_D_ = +0.20° (*c* = 1, MeOH). IR (KBr): 3295, 3065, 2940, 2870, 1640, 1585, 1515, 1460, 1390, 1235, 1025, 880, 740, 695 cm^−1^. ^1^H NMR (500 MHz, DMSOd6): δ = 10.66 (s, 1H, COOH), 7.99 (t, ^3^*J* = 5.4 Hz, 1H, NH), 7.94–7.82 (m, 2H, 2NH), 7.27 (d, ^3^*J* = 7.8 Hz, 1H, H-C(Ar_IND_)), 7.22 (d, ^3^*J* = 8.0 Hz, 1H, H-C(Ar_IND_)), 6.96 (t, ^3^*J* = 7.5 Hz, 1H, H-C(Ar_IND_)), 6.87 (t, ^3^*J* = 7.4 Hz, 1H, H-C(Ar_IND_)), 4.68 (s, 1H, HaC(29)), 4.56 (s, 1H, HbC(29)), 3.76–3.56 (m, 6H, 3H_2_C(CO)), 3.02 (td, ^3^*J* = 10.9, 4.3 Hz, 1H), 2.74 (d, AB syst., ^2^*J* = 14.9 Hz, 1H), 2.62–2.53 (m, 1H), 2.23–2.14 (m, 1H), 2.05 (d, AB syst., ^2^*J* = 15.0 Hz, 1H), 1.94–1.85 (m, 1H), 1.85–1.34 (m, 15H, including s: 1.66, Me), 1.34–0.88 (m, 17H, including 4s: 1.26, 1.16, 0.97, 0.91, 4Me), 0.88–0.81 (m, 1H), 0.78 (s, 3H, Me). ^13^C NMR (126 MHz, DMSOd6) δ = 176.34, 170.87, 169.65, 168.48, 150.96, 141.30, 136.21, 127.66, 119.91, 117.71, 117.27, 110.47, 109.25, 104.87, 54.92, 53.10, 49.62, 48.88, 46.21, 42.16, 42.00, 41.97, 41.92, 40.33, 37.85, 37.59, 36.94, 36.87, 33.96, 33.14, 32.26, 30.41, 30.26, 29.03, 25.39, 22.66, 21.09, 19.05, 18.80, 16.19, 15.55, 14.35. HRMS (ESI): m/z calcd. For [C_42_H_58_N_4_O_5_+H]^+^ 699.4480; found 699.4495 (2.14 ppm) (Supplementary Figures S1–S3). Table 3 contains the trivial name and structural formula of BA1.

BA1-OMe: According to the general method I, compound BA1-Ome was prepared from BA2 (2.6 g, 4.05 mmol, 1 equiv.), EDC·HCl (0.815 g, 4.25 mmol, 1.2 equiv.), HOBt (0.575 g, 4.25 mmo, 1.2 equiv.), DIPEA (0.46 mL, 14.58 mmol, 3.6 equiv.), HCl·H-Gly-Ome (0.11 g, 4.86 mmol, 1.2 equiv.). Purified by column chromatography over SiO_2_ with DCM/i-PrOH (94:6, *v*/*v*). Yield 1.9 g, 66%. Yellow solid powder. [α]_D_ = +0.27° (*c* = 1, CHCl_3_). IR (KBr): 3320, 3070, 2945, 2875, 2840, 1745, 1660, 1510, 1460, 1365, 1210, 1010, 880, 740, 700 cm^−1^. ^1^H NMR (500 MHz, CDCl_3_): δ = 7.79 (br. S, 1H, NH(IND)), 7.38 (d, ^3^*J* = 7.6 Hz, 1H, H-C(Ar_IND_)), 7.28 (d, ^3^*J* = 8.0 Hz, 1H, H-C(Ar_IND_)), 7.15–7.03 (m, 3H, H-C(Ar_IND_), NH), 7.01 (t, ^3^*J* = 5.5 Hz, 1H, NH), 6.63 (t, ^3^*J* = 5.4 Hz, 1H, NH), 4.75 (s, 1H, H_a_C(29)), 4.63 (s, 1H, H_b_C(29)), 4.09–3.90 (m, 6H, 3H_2_C(CO)), 3.75 (s, 3H, MeO), 3.10 (dt, ^3^*J* = 10.9, 4.4 Hz, 1H), 2.82 (d, AB syst., ^2^*J* = 15.0 Hz, 1H), 2.48 (dt, ^3^*J* = 12.2, 3.4 Hz, 1H), 2.13 (d, AB syst., ^2^*J* = 15.0 Hz, 1H), 2.06 (td, ^3^*J* = 13.6, 3.5 Hz, 1H), 1.99–1.75 (m, 3H), 1.75–1.32 (m, 16H, including s: 1.71, Me), 1.32–1.05 (m, 8H, including 2s: 1.28, 1.18, 2Me), 1.03 (s, 3H, Me), 0.98 (s, 3H, Me), 0.85 (s, 3H, Me). ^13^C NMR (126 MHz, CDCl_3_) δ = 178.01, 170.38, 170.24, 169.14, 150.77, 141.02, 136.27, 128.47, 121.07, 119.03, 118.02, 110.46, 109.79, 107.12, 55.99, 53.43, 52.59, 50.22, 49.57, 46.95, 43.76, 43.16, 42.69, 41.29, 40.97, 38.44 (2C), 38.13, 37.42, 34.26, 33.72, 33.66, 30.99, 30.92, 29.76, 25.90, 23.28, 21.64, 19.56, 19.38, 16.51, 15.94, 14.84. HRMS (ESI): m/z calcd. For [C_43_H_60_N_4_O_5_+H]^+^ 713.4636; found 713.4629 (0.98 ppm) (Supplementary Figures S4–S6). Table 3 contains the trivial name and structural formula of BA1-OMe.

BA2: According to general method II, compound BA2 was prepared from BA2-OMe (3.1 g, 4.72 mmol, 1 equiv.), NaOH (0.378 g, 9.45 mmol, 2 equiv.), water (3.1 mL, 166.7 mmol, 35 equiv.). Yield 2.83 g, 93%. Yellow solid powder. [α]_D_ = +0.17° (*c* = 1, MeOH). IR (KBr): 3345, 3065, 2940, 2875, 1640, 1510, 1460, 1390, 1260, 1240, 1095, 1010, 960, 910, 880, 800, 735, 675 cm^−1^. ^1^H NMR (500 MHz, CDCl_3_): δ = 7.71 (br. s, 1H, NH(IND)), 7.38 (d, ^3^*J* = 7.7 Hz, 1H, H-C(Ar_IND_)), 7.28 (d, ^3^*J* = 8.0 Hz, 1H, H-C(Ar_IND_)), 7.17 (t, ^3^*J* = 5.4 Hz, 1H, NH), 7.10 (dd, ^3^*J* = 7.6, 7.3 Hz, 1H, H-C(Ar_IND_)), 7.05 (dd, ^3^*J* = 7.6, 7.2 Hz, 1H, H-C(Ar_IND_)), 6.79 (t, ^3^*J* = 5.8 Hz, 1H, NH), 4.76 (s, 1H, H_a_C(29)), 4.63 (s, 1H, H_b_C(29)), 4.12 (dd, AB syst., ^2^*J* = 18.2 Hz, ^3^*J* = 5.5 Hz, 1H, H_a_C(CO)), 4.03 (dd, AB syst., ^2^*J* = 18.0 Hz, ^3^*J* = 5.2 Hz, 1H, H_b_C(CO)), 4.00 (d, ^3^*J* = 5.4 Hz, 2H, H_2_C(CO)), 3.09 (dt, ^3^*J* = 10.8, 4.2 Hz, 1H), 2.82 (d, AB syst., ^2^*J* = 15.0 Hz, 1H), 2.48 (dt, ^3^*J* = 12.6, 3.0 Hz, 1H), 2.13 (d, AB syst., ^2^*J* = 15.1 Hz, 1H), 2.10–2.03 (m, 1H), 1.99–1.76 (m, 3H), 1.75–1.32 (m, 16H, including s: 1.71, Me), 1.32–1.06 (m, 8H, including 2s: 1.28, 1.17, 2Me), 1.02 (s, 3H, Me), 0.98 (s, 3H, Me), 0.85 (s, 3H, Me). ^13^C NMR (126 MHz, CDCl_3_) δ = 178.36, 172.62, 170.74, 150.75, 141.03, 136.28, 128.47, 121.07, 119.02, 118.02, 110.48, 109.86, 107.08, 56.06, 53.41, 50.15, 49.54, 46.88, 43.21, 42.68, 41.66, 40.96, 38.51, 38.43, 38.09, 37.41, 34.24, 33.67, 33.61, 30.97, 30.89, 29.72, 25.88, 23.27, 21.65, 19.52, 19.38, 16.51, 15.85, 14.84. HRMS (ESI): m/z calcd. for [C_40_H_55_N_3_O_4_+H]^+^ 642.4265; found 642.4261 (0.62 ppm) (Supplementary Figures S7–S9). Table 3 contains the trivial name and structural formula of BA2.

BA2-OMe: According to general method I, compound BA2-OMe was prepared from BA3 (3.25 g, 5.95 mmol, 1 equiv.), EDC·HCl (1.37 g, 7.14 mmol, 1.2 equiv.), HOBt (0.97 g, 7.14 mmo, 1.2 equiv.), DIPEA (3.71 mL, 21.42 mmol, 4.4 equiv.), HCl·H-Gly-OMe (0.90 g, 7.14 mmol, 1.2 equiv.). Purified by column chromatography over SiO_2_ with DCM/i-PrOH (100:3, *v*/*v*). Yield 3.41 g, 87%. Yellow solid powder. [α]_D_ = +0.29° (*c* = 1, CHCl_3_). IR (KBr): 3395, 2945, 2865, 2840, 1745, 1640, 1505, 1460, 1365, 1205, 1010, 880, 735 cm^−1^. ^1^H NMR (500 MHz, CDCl_3_): δ = 7.78 (br. s, 1H, NH(IND)), 7.38 (d, ^3^*J* = 7.6 Hz, 1H, H-C(Ar_IND_)), 7.28 (d, ^3^*J* = 8.0 Hz, 1H, H-C(Ar_IND_)), 7.10 (dd, ^3^*J* = 7.6, 7.3 Hz, 1H, H-C(Ar_IND_)), 7.08–7.01 (m, 2H, H-C(Ar_IND_), NH), 6.53 (t, ^3^*J* = 5.3 Hz, 1H, NH), 4.77 (d, ^4^*J* = 2.3 Hz, 1H, H_a_C(29)), 4.63 (t, ^4^*J* = 1.9 Hz, 1H, H_b_C(29)), 4.09 (dd, AB syst., ^2^*J* = 18.1 Hz, ^3^*J* = 5.6 Hz, 1H, H_a_C(CO)), 4.01 (d, AB syst., ^3^*J* = 5.6 Hz, 1H, H_b_C(CO)), 3.98 (d, ^3^*J* = 5.3 Hz, 2H, H_2_C(CO)), 3.76 (s, 3H, MeO), 3.14 (dt, ^3^*J* = 11.1, 4.4 Hz, 1H), 2.82 (d, AB syst., ^2^*J* = 15.0 Hz, 1H), 2.52 (dt, ^3^*J* = 12.3, 3.4 Hz, 1H), 2.13 (d, AB syst., ^2^*J* = 15.0 Hz, 1H), 2.06 (td, ^3^*J* = 13.6, 3.2 Hz, 1H), 2.01–1.76 (m, 3H), 1.76–1.34 (m, 16H, including s: 1.72, Me), 1.32–1.05 (m, 8H, including 2s: 1.28, 1.39, 2Me), 1.03 (s, 3H, Me), 0.98 (s, 3H, Me), 0.86 (s, 3H, Me). ^13^C NMR (126 MHz, CDCl_3_): δ = 177.62, 170.05, 170.04, 150.86, 141.02, 136.27, 128.46, 121.05, 119.01, 118.02, 110.45, 109.74, 107.12, 56.00, 53.42, 52.54, 50.21, 49.58, 46.87, 43.48, 42.69, 41.33, 40.97, 38.47, 38.44, 38.08, 37.40, 34.26, 33.71, 30.97, 30.91, 29.74, 25.90, 25.50, 23.28, 21.63, 19.57, 19.37, 16.49, 15.92, 14.83. HRMS (ESI): m/z calcd. for [C_41_H_57_N_3_O_4_+H]^+^ 656.4422; found 656.4422 (0 ppm) (Supplementary Figures S10–S12). Table 3 contains the trivial name and structural formula of BA2-OMe.

BA3: According to general method II, compound BA3 was prepared from BA3-OMe (5 g, 8.35 mmol, 1 equiv.), NaOH (0.67 g, 16.70 mmol, 2 equiv.), water (5 mL, 292.2 mmol, 35 equiv.). Yield 3.9 g, 88%. Yellow solid powder. ^1^H and ^13^C NMR spectra matching those reported in the literature [70]. Table 3 contains the trivial name and structural formula of BA3.

BA3-OMe: Compound BA4 (0.5 g, 0.92 mmol, 1 equiv.) was dissolved in anhydrous DCM (5 mL), and few drops of DMF were added. Oxalyl chloride (86 µL, 1.00 mmol, 1.1 equiv.) was added and the reaction was stirred for 1.5 h at room temperature. It was then evaporated and under inert atmosphere redissolved in anhydrous DCM (5 mL). Then Et_3_N (0.4 mL, 2.76 mmol, 1 equiv.) and HCl·H-Gly-OMe (0.173 g, 1.38 mmol, 1.5 equiv.) were added, and the reaction was stirred for 15 h at room temperature. The reaction mixture was diluted with DCM (25 mL) and washed with 50% NaHCO_3_ aq. (3 × 10 mL), brine (10 mL), and dried over Na_2_SO_4_, and the solvent was removed under reduced pressure. The crude product was purified by column chromatography (DCM/i-PrOH (50:1, *v*/*v*). Yield 0.5 g, 91%. Yellow solid powder. ^1^H and ^13^C NMR spectra matching those reported in the literature [50]. Table 3 contains the trivial name and structural formula of BA3-OMe.

BA4: A mixture of betulonic acid (5.0 g, 13.2 mmol, 1 equiv.), phenylhydrazine (4.5 mL, 46.2 mmol, 3.5 equiv.), and glacial acetic acid (60 mL) was heated at reflux for 15 h. After cooling, the reaction mixture was evaporated under reduced pressure. The residue was dissolved in DCM (150 mL), washed with water (4×30 mL), brine (50 mL), dried over Na_2_SO_4_, and the solvent was removed under reduced pressure. The crude product was purified by column chromatography (DCM). Yield 5.0 g, 72%. Yellow solid powder. ^1^H and ^13^C NMR spectra matching those reported in the literature [71]. Table 3 contains the trivial name and structural formula of BA4.

### 4.2. Cell Culture

Human melanoma cell line—A375 (ATCC^®^ CRL-1619^TM^) was acquired from the American Type Culture Collection (ATCC, Manassas, VA, USA); melanoma cells were grown as a monolayer in high glucose Dulbecco’s modified Eagle’s medium (DMEM; Sigma-Aldrich, Taufkirchen, Germany) combined with 10% fetal calf serum (FCS; PromoCell, Heidelberg, Germany) and 1% penicillin/streptomycin mixture (Pen/Strep, 10,000 IU/mL; PromoCell), as previously documented [89]. The cells were maintained under conventional conditions (humidified environment with 5% CO_2_ and 37 °C).

### 4.3. Cellular Viability

The antiproliferative effects of 2,3-indolo-betulinic acid derivatives and betulinic acid were investigated employing the MTT (3-(4,5-dimethylthiazol-2-yl)-2,5-diphenyltetrazolium bromide) assay against A375 human melanoma cells (Sigma-Aldrich, Budapest, Hungary). The experimental studies were performed as previously documented [100]. Succinctly, 1 × 10^4^ cells per well were seeded onto 96-well plates and stimulated with several doses of BA1, BA2, BA3, BA4, BI (1, 10, 25, 50, and 75 µM) under normal parameters (37 °C, 5% CO_2_). Upon 72 h of incubation, 5 mg/mL MTT solution was applied, and the microplates were incubated for a further 4 h. The resultant formazan crystals were dissolved in 100 μL of lysis buffer (Sigma-Aldrich, St. Louis, MO, USA) and the absorbance was captured using a microplate reader (BioRad, xMark Microplate Spectrophotometer) at 570 nm. The control was composed of wells containing untreated cells. The findings are reported as the mean of the three individual investigations. The obtained data were fitted using sigmoidal concentration-response curves, and IC_50_ values were generated using GraphPad Prism (GraphPad Software, San Diego, CA, USA).

### 4.4. Evaluation of the Cytotoxic Potential by Lactate Dehydrogenase (LDH) Assay

The cytotoxic activity of 2,3-indolo-betulinic acid derivatives and betulinic acid on A375 cells was assessed using a lactate dehydrogenase (LDH) method (CyQUANT, Thermo Fisher Scientific, Boston, MA, USA). Various concentrations were utilized, including 1, 10, 25, 50, and 75 µM. The experiment was carried out according to Ghitu et al.’s earlier description [89]. For the experiment, 5000 cells/well were cultivated and allowed to adhere to 96-well culture plates. The cells were treated with the aforementioned doses on a subsequent day and incubated for 72 h. Following 72 h of stimulation, 50 µL from each well was transferred and placed onto a clean 96-well culture plate, combined with 50 µL/well of the reaction mixture, and then incubated for an additional 30 min at room temperature. Subsequently, the stop solution was then applied to each well in a volume of 50 µL. Utilizing an xMark Microplate spectrophotometer (BioRad, xMarkTM Microplate, Serial No. 10578, Tokyo, Japan), at two wavelengths (490 and 680 nm), the amount of LDH released into the medium was measured.

### 4.5. Anti-Migratory Potential—Scratch Assay Method

The scratch assay was performed to examine the regressive influence of the four 2,3-indolo-betulinic acid derivatives and betulinic acid on the invasion potential of the A375 cell line. On 12-well culture plates, 2 × 10^5^ cells/well were placed until 90% proliferation was attained. Subsequently, the adhered cells were scraped using a sterile pipette tip according to the diameter of the well. By gently washing the wells with phosphate-buffered saline, the unattached cells and cellular debris were eliminated. Additionally, 1, 10, 25, 50, and 75 µM doses of the tested compounds were used to stimulate the cells. To assess the cell growth of the treated vs. control (no treatment) cells in the early stages and at regular intervals, wells were photographed at 0 h and 24 h. To facilitate the identification of the same imaging region, a line was placed under each well. For the analysis of cell proliferation, images were captured using an Olympus IX73 inverted microscope equipped with a DP74 camera (Olympus, Tokyo, Japan) and cellSense Dimension software was utilized. To evaluate the migratory capacity of the cells, the percentage of wound closure was determined as stated before [101].

### 4.6. Antimicrobial Activity Assays

The test compounds were investigated for their antimicrobial potential using American Type Culture Collection strains (ThermoScientific, Waltham, MA, USA): *Streptococcus pyogenes* ATCC 19615, *Staphylococcus aureus* ATCC 25923, *Escherichia coli* ATCC 25922, *Pseudomonas aeruginosa* ATCC27853, *Candida albicans* ATCC 10231 and *Candida parapsilosis* ATCC 22019.

The antimicrobial activity of these samples was initially evaluated using the Kirby Bauer disk diffusion method, and when antimicrobial activity was observed, the broth dilution method was used to determine the minimum inhibitory concentration (MIC) and minimum bactericidal concentration (MBC) or minimum fungal concentration (MFC).

All tests were conducted in accordance with the guidelines of the Clinical Laboratory and Standard Institute (CLSI) and the European Committee for Antimicrobial Susceptibility Testing (EUCAST), with minor changes based on the results of our previous investigations [102,103,104,105].

#### 4.6.1. Kirby Bauer Disk Diffusion Method

For each investigated microorganism, suspensions were adjusted to a standardized concentration of 0.5 Mac Farland (approx. 108 colony forming units (CFU)/mL for bacterial strains and 2 × 10^6^ CFU/mL for *Candida* spp.). The agar Mueller–Hinton (MH), MH fastidious (MHF) agar for *Streptococcus pyogenes* and MH–methylene blue agar for *Candida* spp. (bio-Mérieux, Marcy-l’Étoile, France) were inoculated with 100 µL of standardized suspension, then ten microliters from each sample (300 mM) were added to a blank disk (BioMaxima, Poland), and placed on top of the culture medium. The inhibition zones were interpreted based on the positive control (levofloxacin 5 µg and fluconazole 25 µg disk) and the CLSI and National Committee for Clinical Laboratory Standards (NCCLS). All experiments were conducted in triplicate for each strain [105,106].

#### 4.6.2. Broth Dilution Method for Determination of MIC and MBC/MFC

The working microbial suspension (5 × 10^5^ CFU/mL) was prepared by diluting the standard suspension (0.5 Mac Farland). From the stock solutions in dimethyl sulfoxide (DMSO) (1 mM/3 mL), serial dilutions of tested compounds were prepared, and subsequently, 0.1 mL of each diluted solution, 0.4 mL MH or MHF broth, and 0.5 mL microbial suspension were added to four test tubes resulting in the following concentrations for the tested compounds 15, 7.5, 3.7, and 1.8 mM. After incubating the test tubes at 37 °C or 28 °C for 24 h, MIC was determined as the lowest concentration at which observable growth did not occur.

To determine the MBC or MFC, a volume of 1 µL from the tubes with no visible growth was inoculated on Columbia agar + 5% sheep blood or Sabouraud dextrose agar (bio-Mérieux, France) to establish the lowest concentration that killed 99.9% of germs.

### 4.7. Statistical Analysis

Data collection and statistical analysis were performed using GraphPad Prism 8.0.1 (GraphPad Software, San Diego, CA, USA). The data are provided as the mean of three separate experiments ± standard deviation (SD). To assess the statistical differences, one-way ANOVA was utilized, accompanied by Dunnett’s multiple comparisons post-test (* *p* ≤ 0.05; ** *p* ≤ 0.01; *** *p* ≤ 0.001; **** *p* ≤ 0.0001).

## 5. Conclusions

To the best of our knowledge, this is the first in vitro antimelanoma screening of 2,3-indolo-betulinic acid glycine conjugates in the A375 human melanoma cells model and the first screening of their antimicrobial potential. Three out of the four tested indole amides of 2,3-indolo-betulinic acid have attained enhanced anticancer activity when compared to betulinic acid. This statement was underpinned by the antiproliferative, cytotoxic, and anti-migratory capabilities against A375 human melanoma cells. Noteworthy, useful levels of antimelanoma activity were detected and the newly designed *N*-(2,3-indolo-betulinoyl)diglycylglycine (BA1, IC_50_ = 5.7 µM) was superior to the known *N*-(2,3-indolo-betulinoyl)glycine (BA3, IC_50_ = 10.0 µM). This report has also shown that a modification of the peptide side chain in this type of compounds can greatly influence their anticancer activity. We have also revealed that 2,3-indolo-betulinic acid derivatives possess antibacterial activities against Gram-positive bacteria and fungi, with *N*-(2,3-indolo-betulinoyl)glycine (BA3) and 2,3-indolo-betulinic acid (BA4) being the most potent ones. Regarding antifungal activity, both *Candida* spp. strains displayed great sensitivity to BA3. This is preliminary research that may aid in establishing foundational approaches in the fields of oncology and infectious illness. The focus of our future research will be the in-depth exploration of putative mechanisms of action for these derivatives.

## Figures and Tables

**Table 1:** IC_50_ (μM) values yielded from the MTT assay. A375 melanoma cells were incubated with betulinic acid and 2,3-indolo-betulinic acid derivatives for 72 h. The data are presented as the mean ±SD of three replicated assessments.

Compound	IC_50_ (μM)
BI	19.2 ± 0.5
BA1	5.7 ± 0.9
BA2	13.7 ± 0.8
BA3	10.0 ± 0.8
BA4	19.6 ± 0.7

**Table 2:** The diameter of the zone of inhibition, minimum inhibitory concentration, and minimum bactericidal concentration of betulinic acid and 2,3-indolo-betulinic acid derivatives for the investigated strains.

Microbial Strains	Test Compounds	Disk Diffusion Method (Inhibition Zones in mm)	MIC (µg/mL)	MBC or MFC (µg/mL)
*Streptococcus pyogenes* ATCC 19615	BA1 BA2 BA3 BA4 BI Levofloxacin DMSO	9 17 16 17 13 28 9	- 16.33 14.58 13.16 - - -	- 32.66 29.16 26.33 - - -
*Staphylococcus aureus* ATCC 25923	BA1 BA2 BA3 BA4 BI Levofloxacin DMSO	9 17 16 17 9 27 8	- 32.66 29.16 26.33 - - -	- - - - - - -
*Escherichia coli* ATCC 25922	BA1 BA2 BA3 BA4 BI Levofloxacin DMSO	7 7 8 7 7 29 7	- - - - - - -	- - - - - - -
*Pseudomonas aeruginosa* ATCC 27853	BA1 BA2 BA3 BA4 BI Levofloxacin DMSO	7 7 7 7 7 20 7	- - - - - - -	- - - - - - -
*Candida albicans* ATCC 10231	BA1 BA2 BA3 BA4 BI Fluconazole DMSO	9 7 16 7 8 16 8	- - 29.16 - - - -	- - - - - - -
*Candida parapsilosis* ATCC 22019	BA1 BA2 BA3 BA4 BI Fluconazole DMSO	7 7 16 7 9 17 7	- - 29.16 - - - -	- - - - - - -

**Table 3:** Compounds used for the antimelanoma and antibacterial study.

Numbering in the Manuscript	Structural Formula	Trivial Name	CAS Name and Number for Previously Described Compounds
BI	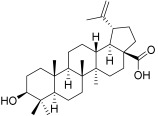	Betulinic acid	3β-Hydroxy-20(29)-lupaene-28-oic acid (472-15-1)
BA1	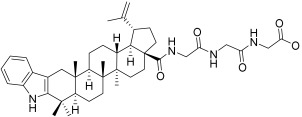	*N*-(2,3-Indolo-betulinoyl)diglycylglycine	-
BA2	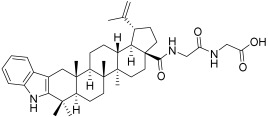	*N*-(2,3-Indolo-betulinoyl)glycylglycine	-
BA3	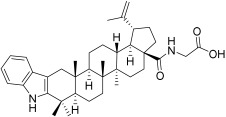	*N*-(2,3-Indolo-betulinoyl)glycine	*N*-(28-Oxo-1′H-lupa-2,20(29)-dieno[3,2-*b*]indol-28-yl)glycine (905838-14-4)
BA4	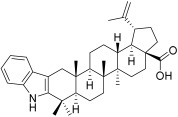	2,3-Indolo-betulinic acid	1’*H*-lupa-2,20(29)-dieno[3,2-*b*]indol-28-oic acid(905837-93-6)

## Data Availability

Data are contained within the article and Supplementary Materials.

## Supplementary Materials

The following supporting information can be downloaded at: https://www.mdpi.com/article/10.3390/plants12061253/s1. Figure S1–S12: NMR spectra of compounds BA1 BA1-OMe, BA2, BA2-OMe. Figure S13: anti-migratory activity of BI and BA1. Figure S14: anti-migratory activity of BA3 and BA3. Figure S15: anti-migratory activity of BA4.
