# Supplementary figures and images for: Comparison of In Vitro Antimelanoma and Antimicrobial Activity of 2,3-Indolo-betulinic Acid and Its Glycine Conjugates
Source: Plants (Basel). 2023 Mar 9;12(6):1253. doi: 10.3390/plants12061253 (PMC10058300; doi:10.3390/plants12061253)

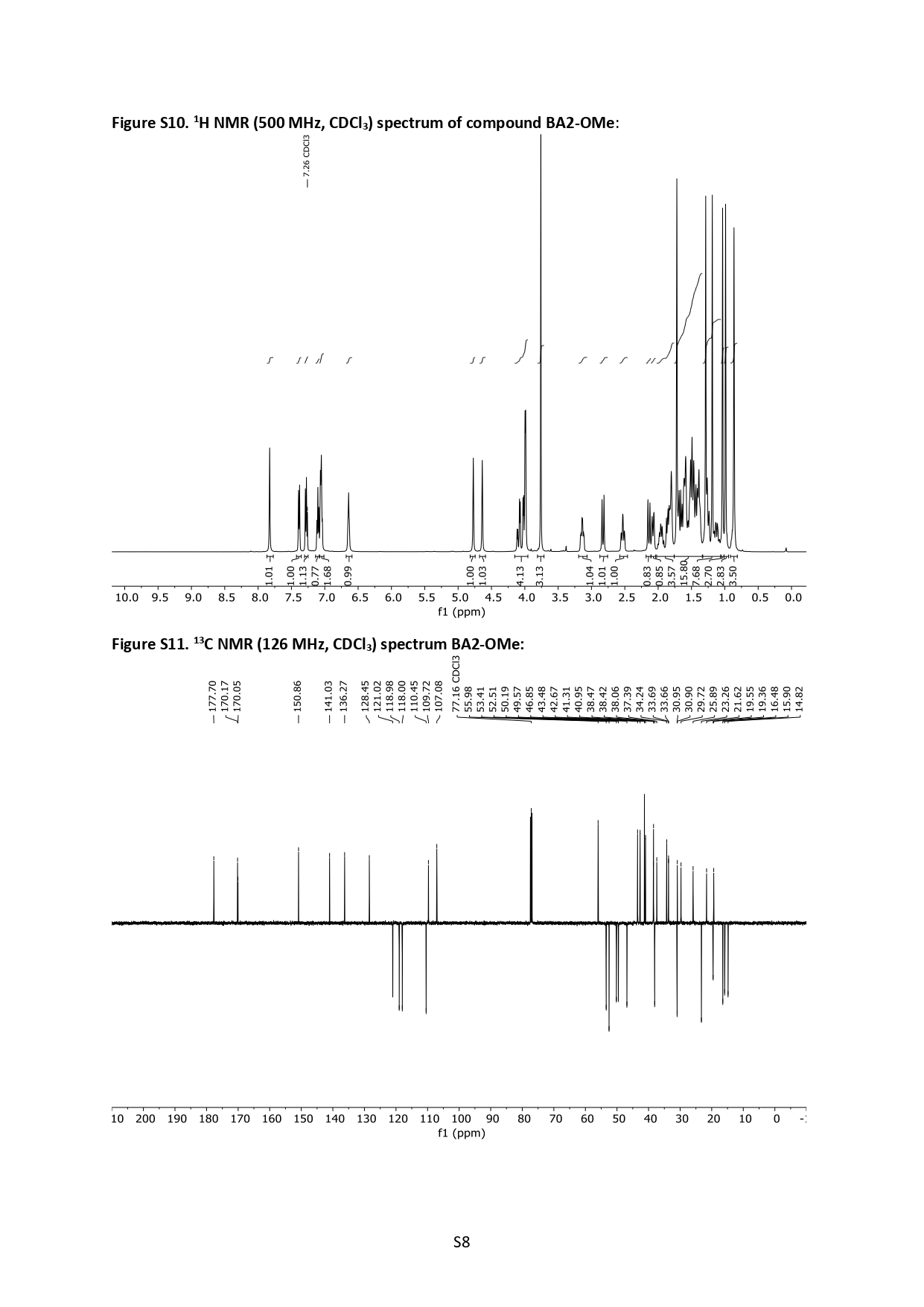

Supplement: Supplementary file 1 [file plants-12-01253-s001.zip › Supplementary figures/Supplementary figure S10-S11.jpg]

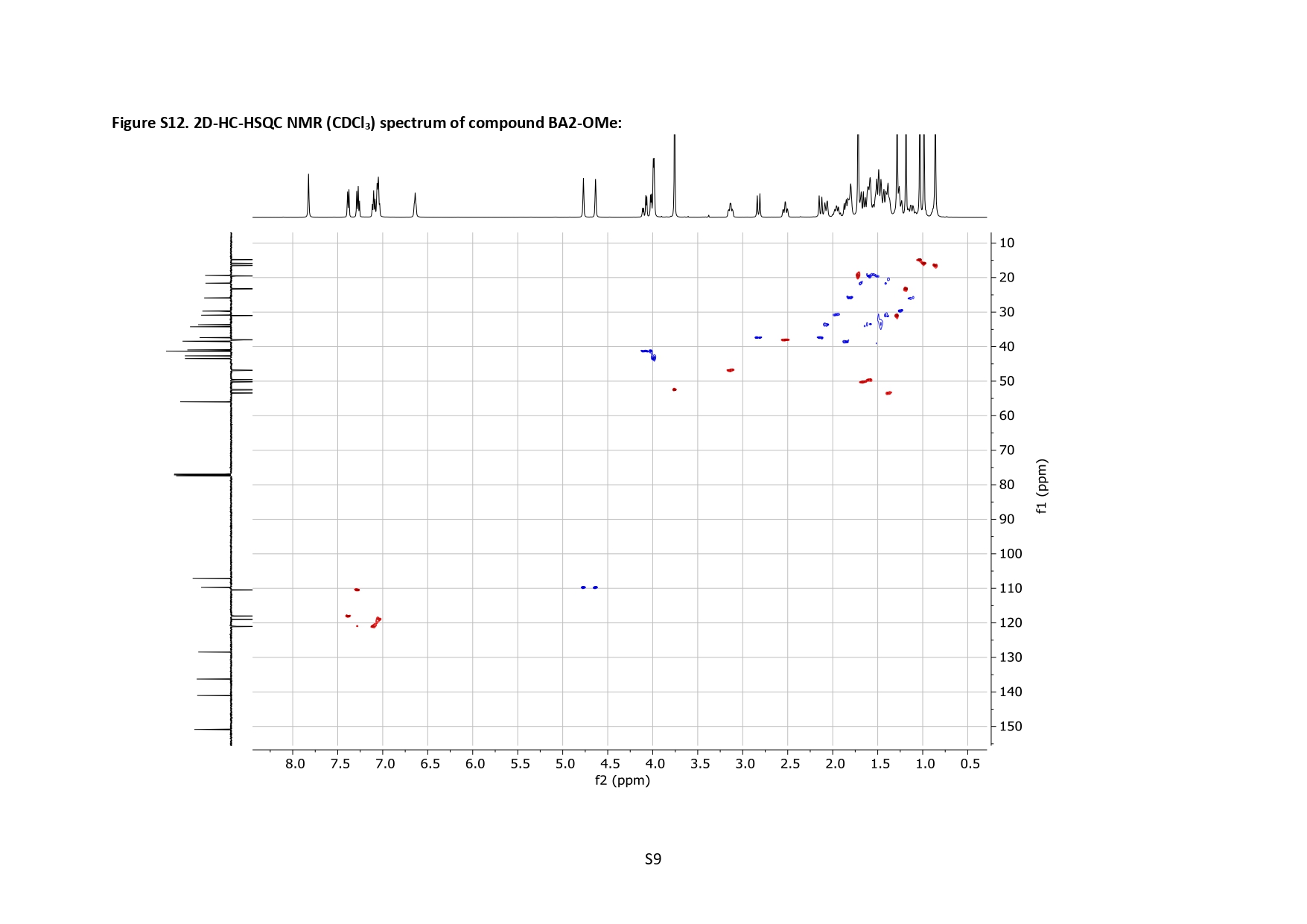

Supplement: Supplementary file 1 [file plants-12-01253-s001.zip › Supplementary figures/Supplementary figure S12.jpg]

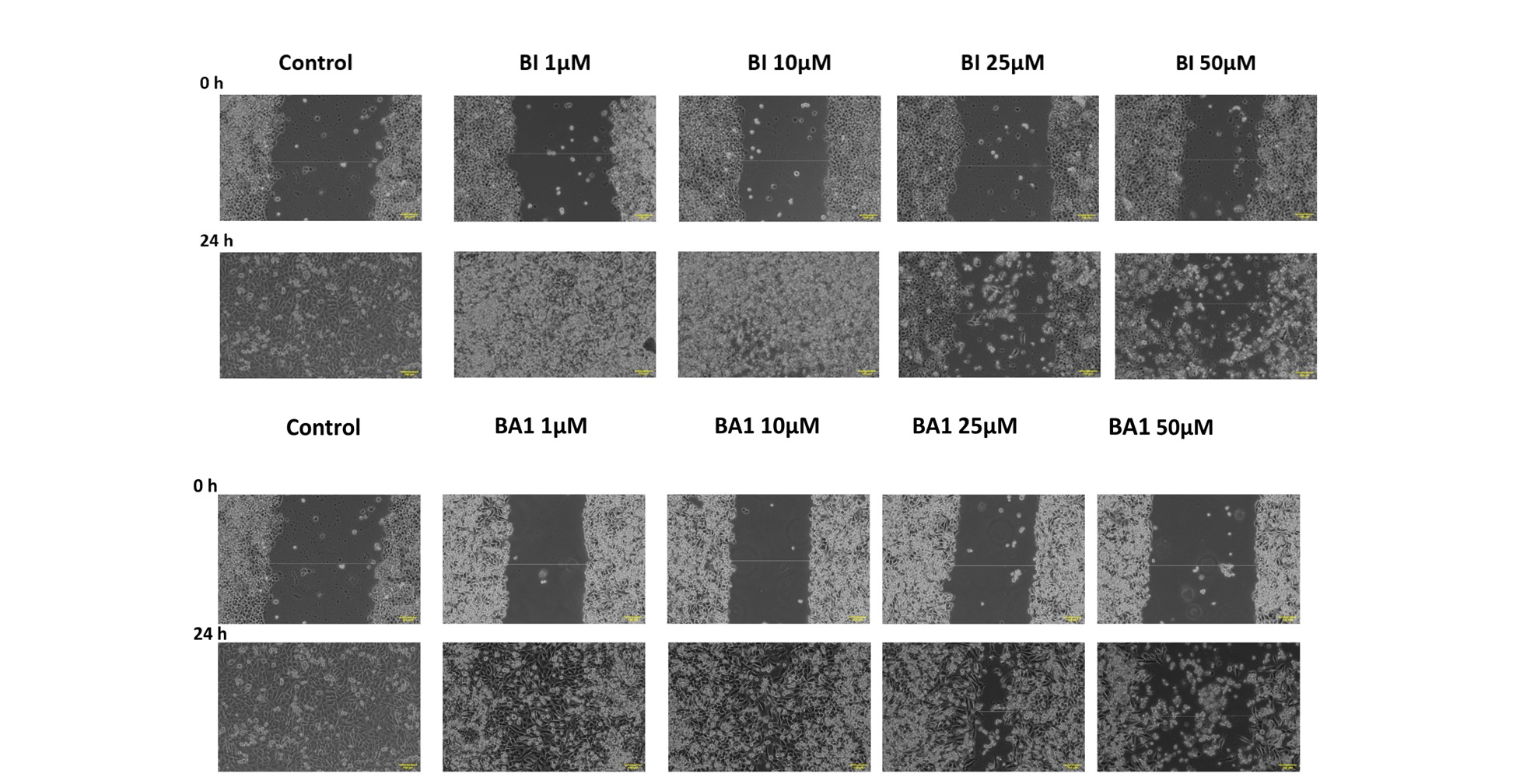

Supplement: Supplementary file 1 [file plants-12-01253-s001.zip › Supplementary figures/Supplementary figure S13.JPG]

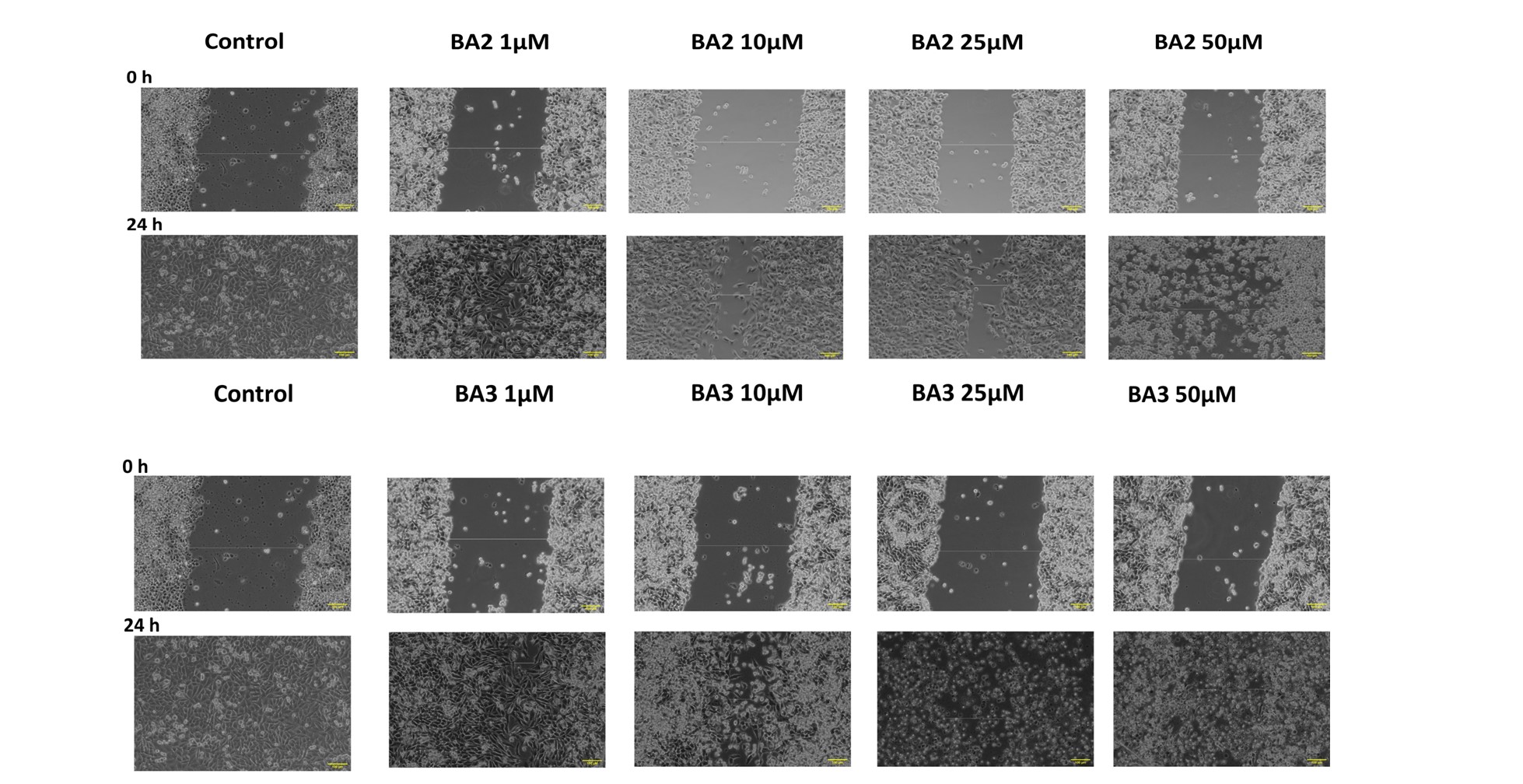

Supplement: Supplementary file 1 [file plants-12-01253-s001.zip › Supplementary figures/Supplementary figure S14.JPG]

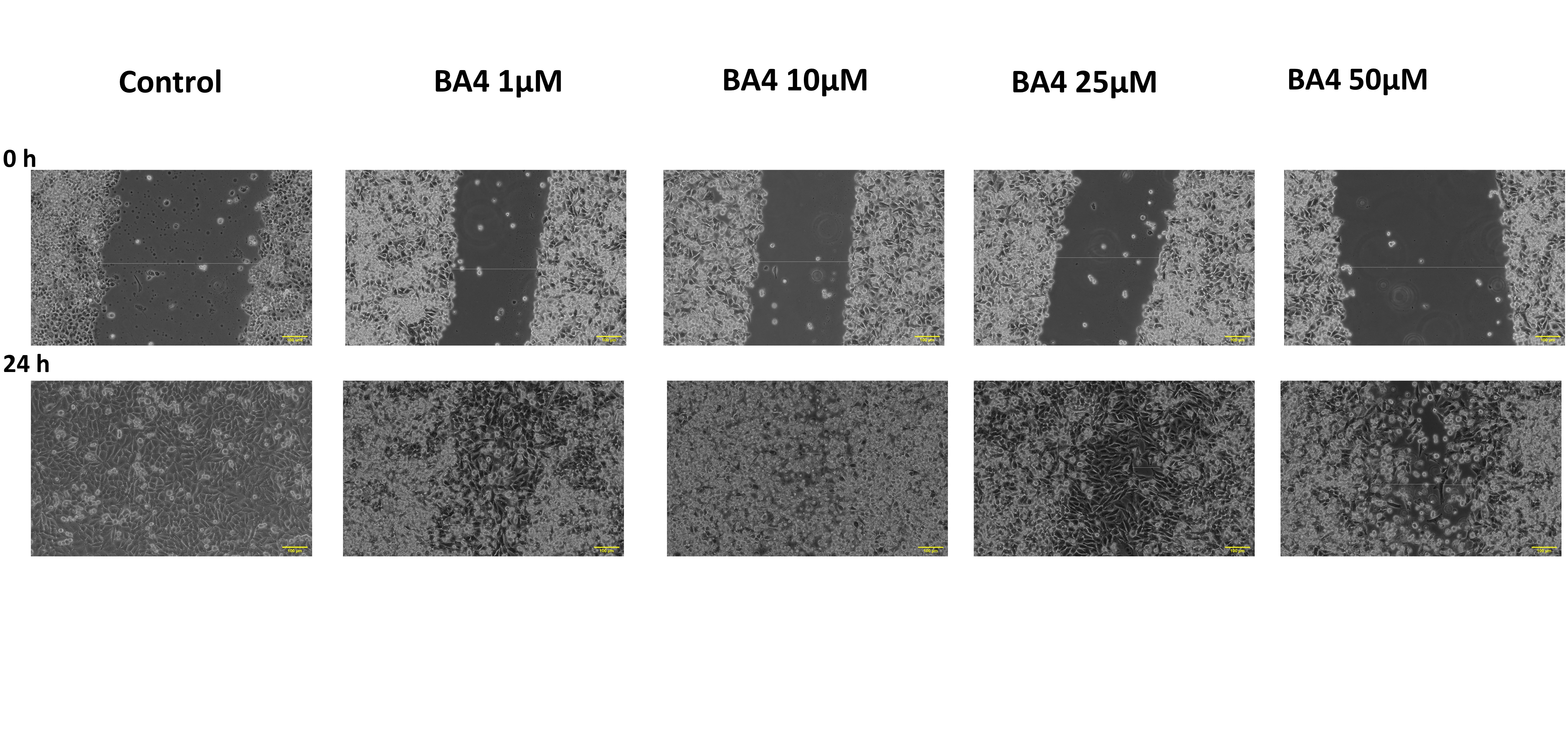

Supplement: Supplementary file 1 [file plants-12-01253-s001.zip › Supplementary figures/Supplementary figure S15.JPG]

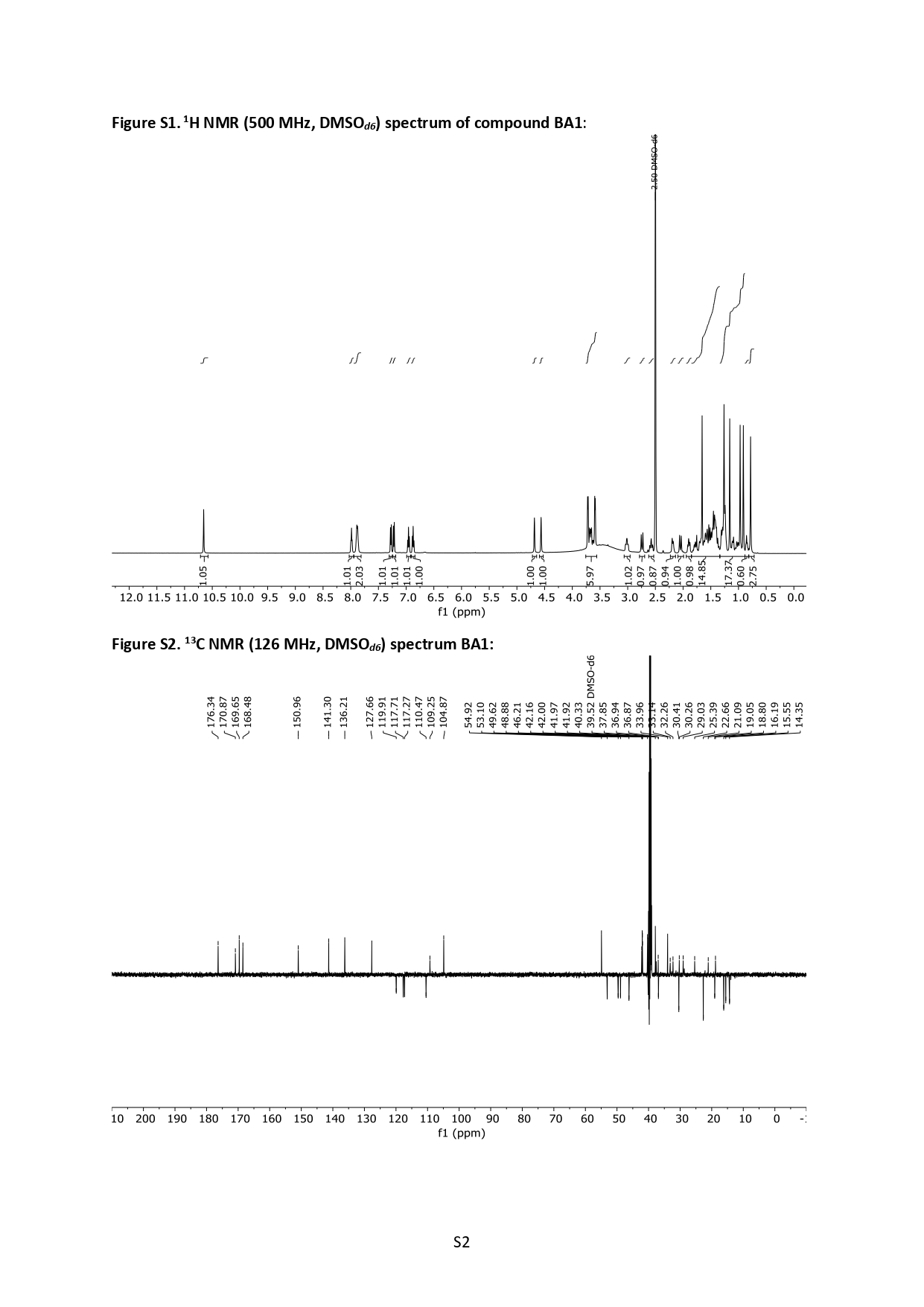

Supplement: Supplementary file 1 [file plants-12-01253-s001.zip › Supplementary figures/Supplementary figure S1-S2.jpg]

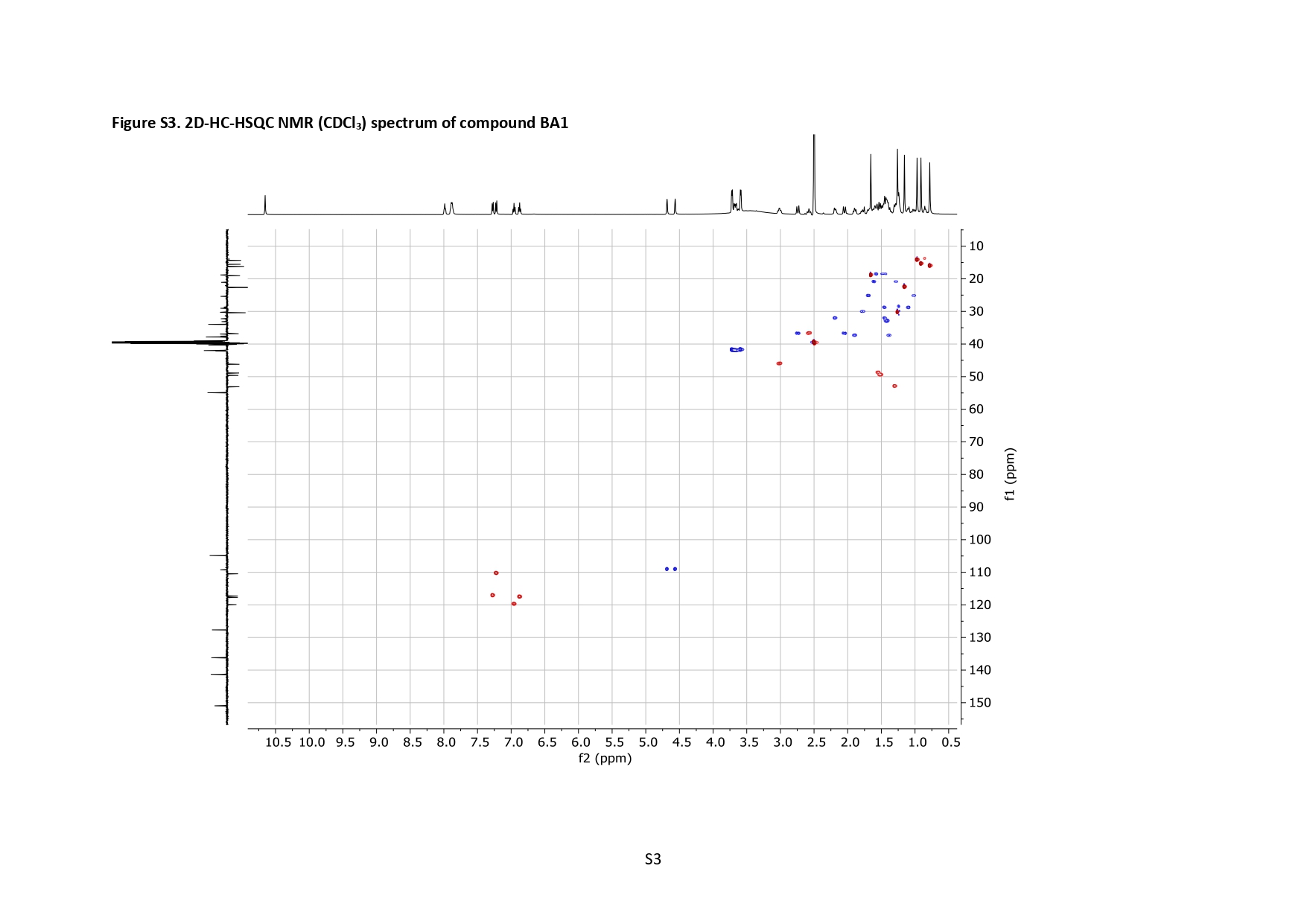

Supplement: Supplementary file 1 [file plants-12-01253-s001.zip › Supplementary figures/Supplementary figure S3.jpg]

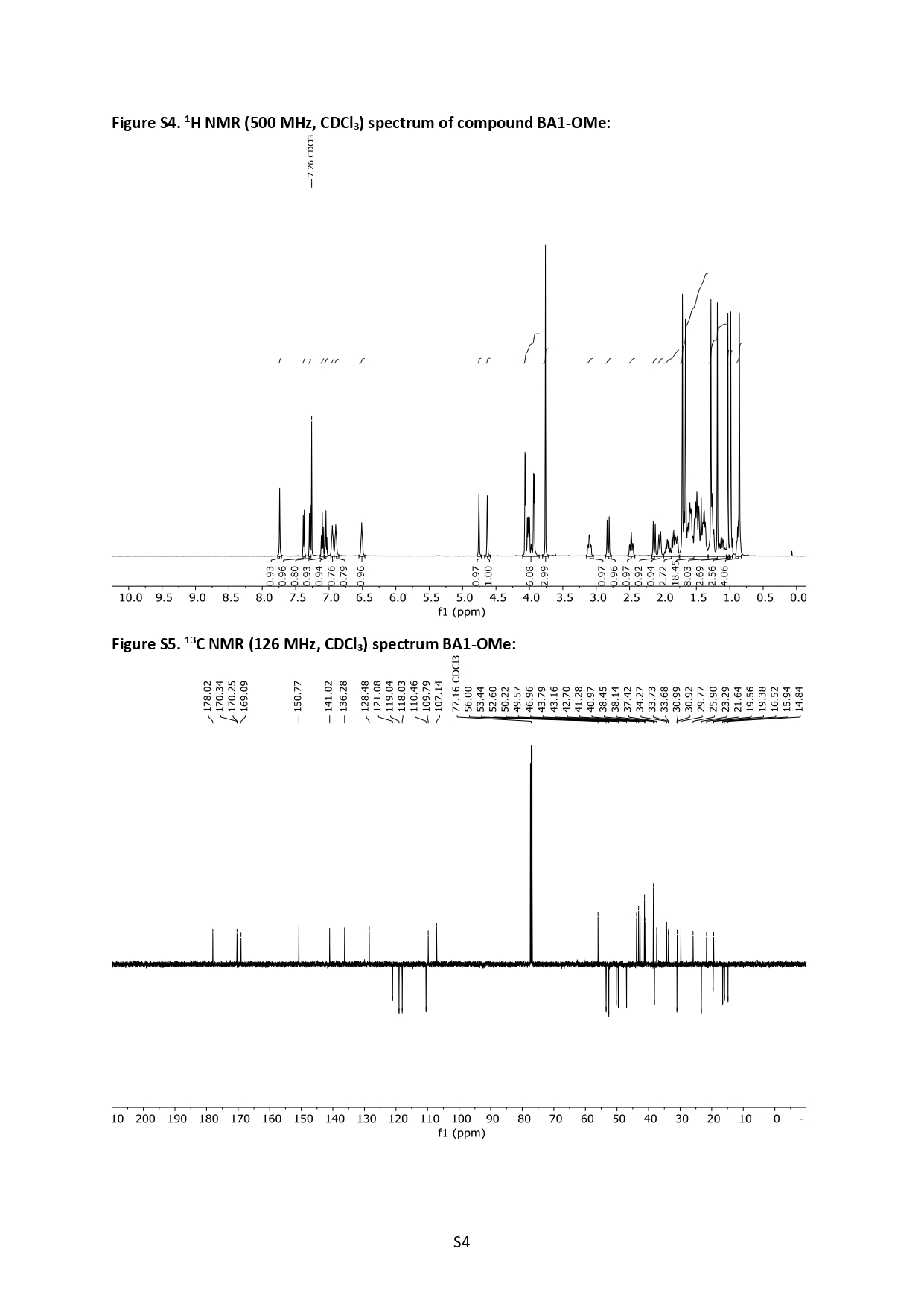

Supplement: Supplementary file 1 [file plants-12-01253-s001.zip › Supplementary figures/Supplementary figure S4-S5.jpg]

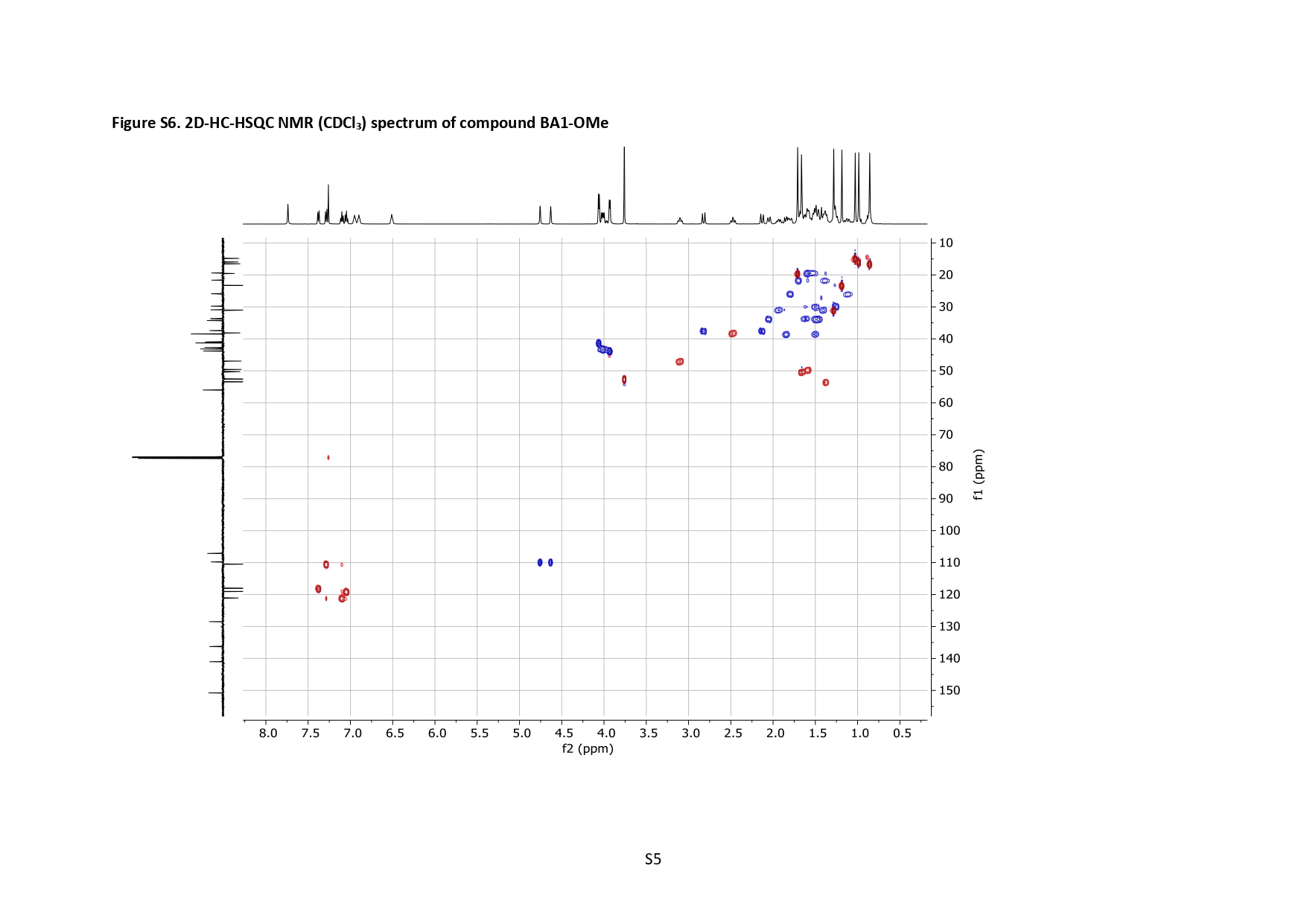

Supplement: Supplementary file 1 [file plants-12-01253-s001.zip › Supplementary figures/Supplementary figure S6.jpg]

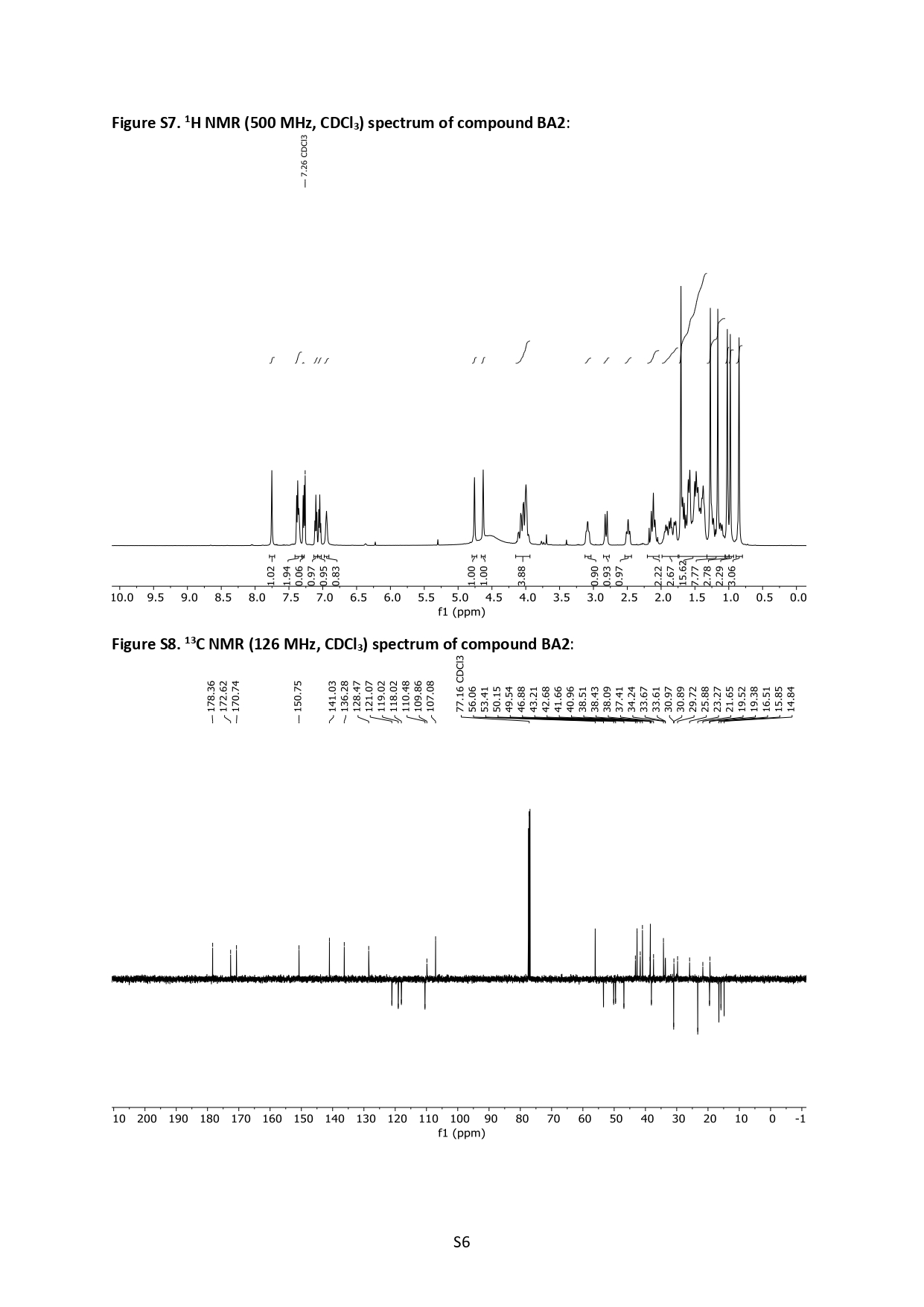

Supplement: Supplementary file 1 [file plants-12-01253-s001.zip › Supplementary figures/Supplementary figure S7-S8.jpg]

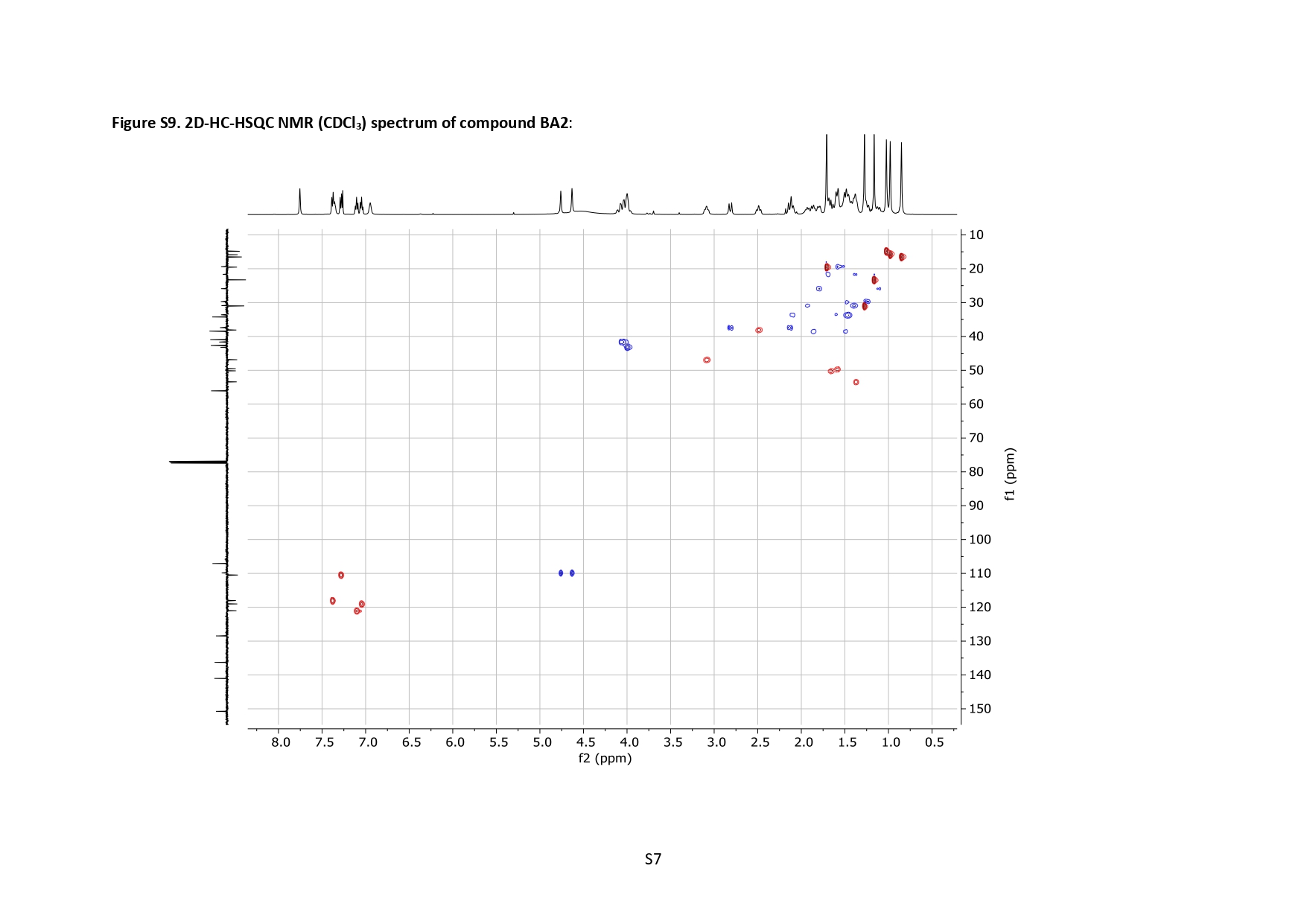

Supplement: Supplementary file 1 [file plants-12-01253-s001.zip › Supplementary figures/Supplementary figure S9.jpg]
